# Microstructural Engineering of Cathode Materials for Advanced Zinc‐Ion Aqueous Batteries

**DOI:** 10.1002/advs.202002722

**Published:** 2020-11-19

**Authors:** Mei Er Pam, Dong Yan, Juezhi Yu, Daliang Fang, Lu Guo, Xue Liang Li, Tian Chen Li, Xunyu Lu, Lay Kee Ang, Rose Amal, Zhaojun Han, Hui Ying Yang

**Affiliations:** ^1^ Pillar of Engineering Product Development Singapore University of Technology and Design 8 Somapah Road Singapore 487372 Singapore; ^2^ Science and Math Cluster Singapore University of Technology and Design (SUTD) 8 Somapah Road Singapore 487372 Singapore; ^3^ School of Chemical Engineering University of New South Wales (UNSW) Kensington New South Wales 2052 Australia; ^4^ CSIRO Manufacturing 36 Bradfield Road Lindfield New South Wales 2070 Australia

**Keywords:** cathode materials, microstructural engineering, zinc‐ion aqueous batteries

## Abstract

Zinc‐ion batteries (ZIBs) have attracted intensive attention due to the low cost, high safety, and abundant resources. However, up to date, challenges still exist in searching for cathode materials with high working potential, excellent electrochemical activity, and good structural stability. To address these challenges, microstructure engineering has been widely investigated to modulate the physical properties of cathode materials, and thus boosts the electrochemical performances of ZIBs. Here, the recent research efforts on the microstructural engineering of various ZIB cathode materials are mainly focused upon, including composition and crystal structure selection, crystal defect engineering, interlayer engineering, and morphology design. The dependency of cathode performance on aqueous electrolyte for ZIB is further discussed. Finally, future perspectives and challenges on microstructure engineering of cathode materials for ZIBs are provided. It is aimed to provide a deep understanding of the microstructure engineering effect on Zn^2+^ storage performance.

## Graphical abstract

Zinc‐ion batteries (ZIBs) have attracted intensive attention due to the low cost, high safety, and abundant resources. Recent advances regarding microstructural engineering on various ZIB cathode materials and the dependency of cathode performance on aqueous electrolyte for ZIBs are summarized. Future perspectives and challenges on microstructure engineering of cathode materials for ZIBs are also provided.

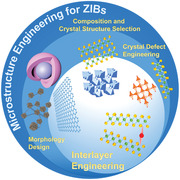

## 1 Introduction

The expanding global demand for potential renewable energy such as wind and solar energy has stimulated the development of efficient and low‐cost electrical energy storage systems. In the 1990s, rechargeable lithium‐ion batteries (LIBs) have been introduced, and gradually dominated the portable electronics and electric vehicle industries.^[^ ^1^, ^2^, ^3^ ^]^ However, limited lithium resources, long‐term potential safety issues, and high cost have greatly impeded the future development of LIBs.^[^ ^1^, ^4^, ^5^, ^6^ ^]^ Owing to the low cost and high safety, aqueous rechargeable batteries have become one of the promising next‐generation energy storage systems for the rapidly growing markets of portable electronics and electric vehicles.^[^ ^7^, ^8^, ^9^ ^]^ In addition to that, aqueous batteries with water‐based electrolytes exhibit much higher ionic conductivity as compared to that of nonaqueous electrolytes, further enabling high rate capability.^[^ ^10^, ^11^, ^12^ ^]^

Among the various aqueous‐based batteries, zinc‐ion batteries (ZIBs) with mild neutral pH or slightly acidic electrolyte have attracted intensive attention owing to their low redox potential, high theoretical volumetric energy density, and good stability in water.^[^ ^10^, ^13^, ^14^ ^]^ Besides, high capacity and energy density can be achieved in ZIBs due to the involvement of two electron transfer in the electrochemical reaction of divalent Zn^2+^.^[^ ^15^ ^]^ To date, intensive efforts have been done to explore suitable cathode materials for aqueous ZIBs.^[^ ^9^, ^10^, ^14^, ^16^ ^]^ However, the high polarization property of Zn^2+^ induces a strong electrostatic interaction between Zn^2+^ and cathode hosts, leading to difficulty in Zn^2+^ diffusion and insertion/extraction.^[^ ^9^, ^10^ ^]^ As a consequence, the well‐investigated insertion/extraction hosts for Li^+^ and Na^+^ generally demonstrate a low capacity and poor cycling performance in ZIBs. Moreover, the fundamental understanding of the reaction mechanisms for cathode materials in aqueous ZIBs remains controversial, imposing great challenges to motivate the full potential of aqueous ZIBs.^[^ ^10^, ^14^, ^16^ ^]^

To solve the abovementioned challenges, microstructure engineering of cathode materials has received a lot of interest in pursuing suitable cathode materials for ZIBs.^[^ ^9^, ^10^, ^13^, ^16^ ^]^ Microstructure engineering of materials, taking the material composition/crystal structure, crystal defect, interlayer spacing, and morphology into account, is an effective way to boost electrochemical performances of cathode materials. More specifically, intensive studies have revealed that the crystallographic forms of cathode materials play an important role in governing the reaction mechanism in aqueous ZIBs.^[^ ^1^, ^10^, ^13^, ^14^ ^]^ For example, various reaction mechanisms such as Zn^2+^ insertion/extraction, conversion reaction, and Zn^2+^/H^+^ coinsertion/extraction are observed for manganese dioxide (MnO_2_) polymorphs with different tunneled, layered, or 3D structures.^[^ ^9^, ^10^, ^14^, ^16^ ^]^ Furthermore, defect engineering has recently been reported as an effective approach to promote the Zn^2+^ insertion/extraction ability in the current existing cathode materials by fine‐tuning of the active sites and Zn^2+^ interactions.^[^ ^17^, ^18^, ^19^, ^20^ ^]^ Besides, interlayer engineering is another important approach for tuning interlayer properties in layered materials, which modulates their Zn^2+^ insertion/extraction abilities.^[^ ^21^, ^22^ ^]^ Specifically, interlayer expansion can effectively promote diffusion kinetics by reducing the diffusion barrier, thereby converting intrinsically inactive insertion/extraction host into efficient Zn^2+^ storage materials. Moreover, morphology engineering has been devoted to functionalizing a cathode material in terms of superior rate capability, long cycling performance, and high specific capacity for ZIBs.^[^ ^23^, ^24^, ^25^, ^26^ ^]^ The integration of active materials into functionalized microstructure morphology will surely benefit to unlock new material functionalities in typical cathode materials for governing their electrochemical performances, owing to the synergy effects from each design structure advantages.

**Figure 1:**
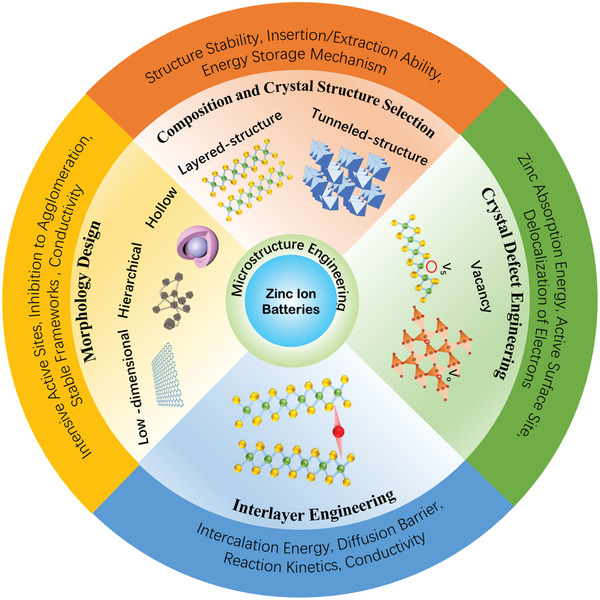
Schematic illustration of microstructure engineering for the cathode in ZIBs.

While the above examples indicate that microstructure engineering plays an important role in modulating properties and improving performance, up to date, a comprehensive review on the microstructure engineering of cathode materials to improve their electrochemical performance in aqueous ZIBs is still lacking. In this review, we mainly focus on the recent significant advances in the microstructure engineering of cathode materials for advanced aqueous ZIBs. We will first briefly highlight the current issues of cathode materials for aqueous ZIBs, including the lack of suitable Zn^2+^ insertion/extraction materials and the involvement of complex and controversial energy storage mechanisms. The microstructure engineering approaches of cathode materials for achieving high electrochemical performance in aqueous ZIBs are then discussed, including composition and crystal structure selection, crystal defect engineering, interlayer engineering, and morphology design (**Figure** **1**). The electrolytes and their effects on the zinc storage mechanism will also be discussed since they play a critical role in achieving high‐performance aqueous ZIBs. Finally, a brief conclusion and perspective on the microstructure engineering of cathode materials for further research efforts in aqueous ZIBs will be presented.

## 2 Current Issues of Cathode

### 2.1 The Lack of Suitable Zn^2+^ Insertion/Extraction Cathode Materials

The lack of suitable Zn^2+^ insertion/extraction cathode materials with high reversible capacity, excellent cycling performance, and adequate operating voltage is a critical issue in aqueous ZIBs. Although the ionic size of Zn^2+^ (139 pm) is smaller than that of Li^+^ (182 pm) and Na^+^ (227 pm), the high polarization property of Zn^2+^ has induced a strong interaction with water molecules, resulting in the formation of a large clathrate structure with a size of 5.5 Å in aqueous electrolyte.^[^ ^27^ ^]^ The formation of the large clathrate structure will further induce difficulty in Zn^2+^ diffusion and storage, leading to the accumulation of Zn^2+^ in the lattice and thus the structural transformation and disproportional reaction during cycling. Therefore, typical cathode materials suitable for Li^+^ insertion/extraction such as layered oxides and transition metal chalcogenides, generally experience sluggish ion kinetics in ZIBs with low capacity.^[^ ^27^, ^28^ ^]^ Manganese oxide and vanadium oxide have been widely studied as potential Zn^2+^ insertion/extraction hosts, but the cathode dissolution and the formation of irreversible discharged by‐product associated with the structural transformation and disproportional reaction during cycling, greatly degrade the cycling stability.^[^ ^7^, ^29^, ^30^, ^31^ ^]^ The gradual precipitation/dissolution during cycling may further result in a low active material utilization efficiency, inhibition of the ion transportation, and high electrochemical impedance.^[^ ^32^ ^]^ Therefore, it is crucial to explore the novel design of new cathode materials with high electrochemical activity in ZIBs, excellent structural integrity, little side reaction, and high electrical conductivity simultaneously for boosting the electrochemical performances of ZIBs.

### 2.2 Complex and Controversial Reaction Mechanisms

While there has been much significant progress in the advancement of ZIB performance over the past few years, the reaction mechanisms of various cathodes in aqueous ZIBs are not fully understood and established. Different from the monovalent‐alkali‐metal‐ion‐based working mechanisms, the mechanisms in aqueous ZIBs are complicated and debatable. According to the literature, the reaction mechanisms of aqueous ZIBs are mainly categorized into Zn^2+^ insertion/extraction mechanism, conversion reaction, and H^+^ and Zn^2+^ coinsertion/extraction.^[^ ^10^, ^11^, ^14^ ^]^ In addition to the aforementioned energy storage mechanisms, surface redox reactions and phase transition are also observed in aqueous ZIBs and contribute additional capacity during cycling.^[^ ^34^, ^35^ ^]^

Owing to the small ionic radius of Zn^2+^ (0.74 Å), various compounds with tunneled and layered structures allow the insertion/extraction of Zn^2+^. For example, Kang and co‐workers proposed the electrochemical reaction mechanism in the aqueous Zn/*α*‐MnO_2_ system was based on the reversible insertion/extraction of Zn^2+^ as below^[^ ^7^ ^]^

(1) $$\begin{array}{c} \mathrm{Cathode}:\;Zn^{2+}+2e^{-}+2\alpha -\mathrm{Mn}O_{2}↔\mathrm{ZnM}n_{2}O_{4} \end{array}$$

(2) $$\begin{array}{c} \mathrm{Anode}:\;\mathrm{Zn}↔Zn^{2+}+2e^{-} \end{array}$$

Their following works further demonstrated that the main charge/discharge mechanism for other tunneled‐structure MnO_2_ such as *β*‐, *γ*‐, and *δ*‐type MnO_2_ was based on the reversible Zn^2+^ insertion/extraction.^[^ ^36^ ^]^ It is worth noting that some studies have revealed the phase transformation in *α*‐MnO_2_ during insertion/extraction of Zn^2+^.^[^ ^37^, ^38^ ^]^ For example, Lee et al. reported a phase transition from tunneled MnO_2_ to layered Zn‐buserite, instead of spinel ZnMn_2_O_4_. Later, Kim and co‐workers further proposed a complex multiphase transformation during the insertion of Zn^2+^ in *γ*‐MnO_2_ for an aqueous Zn/*γ*‐MnO_2_ cell, involving spinel‐type ZnMnO_4_, tunnel‐type Zn*_x_*MnO_2_, and a layered‐type Zn*_x_*MnO_2_.^[^ ^39^ ^]^ It is worth noting that the Zn‐insertion behaviors in aqueous ZIBs are different among MnO_2_ polymorphs such as *γ*‐, *α*‐, and *β*‐MnO_2_.^[^ ^38^, ^39^, ^40^, ^41^ ^]^ The different insertion/extraction behaviors of Zn^2+^ in MnO_2_ polymorphs may have corresponded to the differences in their microstructure properties such as crystal structure arrangements that modulate the ion insertion/extraction thermodynamics and kinetics.^[^ ^42^ ^]^

Other materials such as V_2_O_5_ and MoS_2_ with layered structure have also been investigated to undergo the Zn^2+^ insertion/extraction reaction mechanism.^[^ ^27^, ^43^ ^]^ For example, Liang et al. reported a layered oxygen‐incorporated MoS_2_ (O‐MoS_2_) cathode, which enables high reversibility for hydrated Zn^2+^ insertion/extraction into/from the layered host accompanied by the reversible 2H to 1T MoS_2_ phase transition.^[^ ^27^ ^]^ It is worth to note that the reversible surface Mo^4+^/Mo^6+^ redox reaction also contributes to the capacity. Owing to their open framework, Prussian blue analogs (PBAs)^[^ ^44^, ^45^ ^]^ and polyanion‐based^[^ ^46^, ^47^ ^]^ materials have been reported to allow the insertion/extraction of Zn^2+^. For example, Zhang et al. reported the energy storage mechanism of the Zn/zinc hexacyanoferrate system involving the insertion/extraction of Zn^2+^ at the zinc hexacyanoferrate cathode, and deposition/dissolution of zinc at the zinc anode.

It is worth to point out that a different reaction mechanism, which is based on the conversion reaction between *α*‐MnO_2_ and MnOOH for a Zn/*α*‐MnO_2_ system with preaddition of Mn^2+^ in the electrolyte, has been reported.^[^ ^30^ ^]^ They revealed the formation of MnOOH at the fully discharged state, which is due to the reaction between MnO_2_ and a proton from water. To achieve a neutral charge in the system, the OH^−^ reacts with the aqueous electrolyte to form ZnSO_4_[Zn(OH)_2_]_3_. They have proposed the reaction mechanisms as below

(3) $$\begin{array}{c} \mathrm{Cathode}:H_{2}O↔H^{+}+OH^{-} \end{array}$$

(4) $$\begin{array}{c} \alpha -\mathrm{Mn}O_{2}+H^{+}+e^{-}↔\mathrm{MnOOH} \end{array}$$

(5) $$\begin{array}{c} \frac{1}{2}Zn^{2+}+OH^{-}+\frac{1}{6}\mathrm{ZnS}O_{4}+\frac{x}{6}H_{2}O↔\frac{1}{6}\mathrm{ZnS}O_{4}{\left[\mathrm{Zn}{\left(\mathrm{OH}\right)}_{2}\right]}_{3}\cdot xH_{2}O \end{array}$$

(6) $$\begin{array}{c} \mathrm{Anode}:\frac{1}{2}\mathrm{Zn}↔\frac{1}{2}Zn^{2+}+e^{-} \end{array}$$

It is important to note that the recent works have further revealed that mechanism in the Zn/MnO_2_ system with preaddition of Mn^2+^ in the electrolyte is dynamic and the phase transformation at MnO_2_ cathode is irreversible during cycling.^[^ ^48^ ^]^ More importantly, they found out that Mn^2+^ in the electrolyte takes part in the reaction. In detail, Zn*_x_*MnO_2_, MnOOH, Mn_2_O_3_, and by‐product ZnSO_4_·3Zn(OH)_2_·5H_2_O are formed in the first discharge process, followed by the formation of *α*‐MnO_2_ and ZnMn_3_O_7_·3H_2_O in the first charge process. During cycling, ZnMn_2_O_4_ and ZnMn_3_O_8_ are further formed on the surface of MnO_2_ and act as the hosts for Zn^2+^ insertion.

Moreover, another electrochemical mechanism of H^+^ and Zn^2+^ insertion/extraction has also been reported for the feasible cathode host materials with an open tunnel or layered framework.^[^ ^49^, ^50^ ^]^ Owing to the observed significant difference in thermodynamics and kinetics between the two reaction regions by the capacitance voltage (CV) sweeps in various rates, galvanostatic intermittent titration technique (GITT) and electrochemical impedance spectroscopy (EIS) analysis, Sun et al. proposed a consequent H^+^ and Zn^2+^ insertion/extraction mechanism for Zn/*α*‐MnO_2_ system.^[^ ^49^ ^]^ The consecutive presence of the MnOOH and ZnMn_2_O_4_ diffraction peaks in ex situ X‐ray diffraction (XRD) analysis strongly confirms that the Zn/*α*‐MnO_2_ system undergoes H^+^ insertion followed by Zn^2+^ insertion. Simultaneous insertion of H^+^ and Zn^2+^ mechanism has also been reported in ZIBs to achieve enhanced synergistic effect of their ion insertion thermodynamics and kinetics. For example, Wan et al. reported NaV_3_O_8_·1.5H_2_O nanosheets with a large interlayer spacing (0.708 nm) as an ideal cathode of ZIBs that enables the simultaneous insertion/extraction of H^+^ and Zn^2+^ into/from the V_3_O_8_ layer.^[^ ^50^ ^]^ They further revealed that the reaction mechanism was different from the consequent insertion/extraction mechanism of H^+^ and Zn^2+^ into/from MnO_2_, where the insertion/extraction of Zn^2+^ and H^+^ involved a two‐step process.^[^ ^49^ ^]^ In detail, the H^+^ and Zn^2+^ simultaneously inserted into NaV_3_O_8_ to form H_3.9_NaZn_0.5_V_3_O_8_·1.5H_2_O in the first discharge process. This process was not a completely reversible reaction. After charging to 1.25 V, the H^+^ and partial Zn^2+^ were simultaneously extracted to form NaZn_0.1_V_3_O_8_·1.5H_2_O, which was a reversible process.

In addition to the aforementioned mechanisms, surface redox reactions have been proposed in a few cathode materials.^[^ ^34^, ^35^ ^]^ For example, Wan et al. demonstrated surface redox reactions including the vanadium redox process and oxygen redox chemistry as the main energy storage mechanisms for Zn/layered VOPO_4_ system.^[^ ^35^ ^]^ Besides, Fang et al. have recently revealed that the energy storage mechanisms of vanadium nitride were based on not only the typical cationic (Zn^2+^/H^+^) insertion/extraction, but also the cationic redox reaction (V^3+^ ↔ V^2+^) and the release/uptake of anions on the vanadium nitride surface with reversible anionic redox reaction (N^3−^ ↔ N^2−^).^[^ ^34^ ^]^ Zhi and co‐workers have further revealed an unusual electrochemical behavior in MXene oxidation derivatives in an aqueous Zn/V_2_CT*_x_* MXene system.^[^ ^23^ ^]^ They found that the delamination of the V_2_CT*_x_* MXene and the gradual phase transition of the V_2_CT*_x_* MXene to V_2_O_5_ were the main contributions of the unusual capacity enhancement. It is worth mentioning that the phase transition behavior of electrodes generally results in performance degradation, showing not favorable for any energy storage system. Nonetheless, Zhi and co‐workers have demonstrated that the outstanding cycle stability and excellent specific capacity can be achieved in an aqueous Zn/V_2_CT*_x_* MXene system.

Overall, different reaction mechanisms in ZIB cathodes have been proposed. These differences may be correlated to their various microstructure properties such as crystal structure arrangements, crystal defects, interlayer properties, and morphologies, which are highly related to the ion insertion thermodynamics, kinetics of H^+^/Zn^2+^, and surface redox reaction. Therefore, microstructure engineering of a cathode material plays an important role in determining and regulating the energy storage reaction mechanism in aqueous ZIBs.

## 3 Microstructure Engineering for High Performance Zinc Cathodes

Properties of functional materials are highly dependent on their microstructures. Therefore, to design a cathode material for aqueous ZIBs, it is important to understand the relationship between microstructures and their electrochemical performance. Microstructure engineering is an effective approach to functionalize a material by the configuration of its internal components, which can modulate the mechanical, chemical, and electrical properties of engineered materials, delivering potential candidates as cathode materials for ZIBs. Herein, we focus on the four aspects of microstructure engineering, including composition and crystal structure selection, crystal defect engineering, interlayer engineering, and morphology design to promote the electrochemical performance of cathode materials for ZIBs.

### 3.1 Composition and Crystal Structure Selection

The electrochemical properties of cathodes are usually governed by the intrinsic nature of the selected materials. The intrinsic nature properties such as composition and crystal structure arrangement exhibit significant effects on the chemical, electronic, electrochemical, and physical properties of a material. This will further result in significant variations in their electrochemical performance for the same type of cathode materials. Therefore, understanding the fundamental chemistry of cathode material composition and crystal structure is critical to provide a general guide for the construction of high‐performance ZIB cathodes.

#### 3.1.1 Manganese‐Based Materials

To date, the development of suitable insertion/extraction host materials for aqueous ZIBs is still in its infancy. Intensive efforts are devoted to searching for crystal structures that can readily accommodate Zn^2+^ and exhibit a highly reversible insertion/extraction structure during the cycling process.

**Figure 2:**
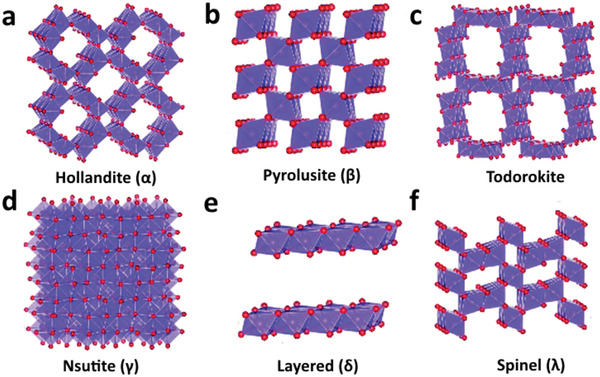
The widely investigated manganese oxide polymorphs for ZIBs: a) Hollandite, b) Pyrolusite, c) Todorokite, d) Nsutite, e) Layered, f) Spinel. Reproduced with permission.^[^ ^16^ ^]^ Copyright 2019, The Royal Society of Chemistry.

Owing to the low cost, moderate operating voltage, and a high theoretical capacity of ≈308 mAh g^−1^, Mn‐based materials are widely investigated as cathode materials for aqueous ZIBs.^[^ ^13^ ^]^ Up to date, MnO_2_ is the most commonly investigated cathode material for ZIBs due to its diversity of crystal structures and multivalent states of manganese. Manganese dioxide possesses a basic crystal structure with an octahedral unit comprised of six oxygen atoms and one manganese atom. Each MnO_6_ octahedral unit can be interconnected by sharing corners/edge into tunneled, layered, or 3D‐type structures.^[^ ^51^ ^]^ Generally, different polymorphs of manganese dioxide are formed through various corner/edge sharing of fundamental MnO_6_ octahedra. They are classified into 1) tunnel‐based, 2) layered‐based, and 3) spinel‐based structures of MnO_2_. The widely investigated tunnel‐based MnO_2_ structures for ZIBs consist of: i) hollandite‐type MnO_2_ (*α*‐MnO_2_) which has a large 2 × 2 tunnel structure with four corner‐sharing MnO_6_ octahedra (**Figure** **2**a); ii) pyrolusite‐type MnO_2_ (*β*‐MnO_2_) which comprises of a 1 × 1 tunnel framework structure by sharing corner (Figure 2b); iii) todorokite‐type MnO_2_ which comprises of large 3 × 3 tunnels with the same structure as *α*‐MnO_2_ (Figure 2c); and iv) Nsutite‐type (*γ*‐MnO_2_) which is the intergrowth structures of 1 × 1 and 1 × 2 tunnels (Figure 2d). Other polymorphs such as v) layered‐type MnO_2_ (*δ*‐MnO_2_) which is constructed by 2D infinite MnO_6_ octahedra sheets with a large interlayer spacing (Figure 2e) and vi) spinel‐type (*λ*‐MnO_2_) which is comprised of 3D spinel structure with Mn^2+^ in the tetrahedral sites and Mn^3+^ in the octahedral sites (Figure 2f), have also been reported to exhibit the ability to allow the insertion/extraction of Zn^2+^.

By analyzing the electrochemical performance based on the crystal structure of MnO_2_ polymorphs, tunnel‐based *α*‐MnO_2_ (2 × 2 tunnels, ≈4.6 Å), *γ*‐MnO_2_ (1 × 1, 2.3 × 2.3 and 1 × 2, 2.3 × 4.6 Å), and layered‐based *δ*‐MnO_2_, are proven to be potential cathode materials for aqueous ZIBs due to their capability of Zn^2+^ insertion/extraction.^[^ ^7^, ^39^, ^52^, ^53^ ^]^ While *β*‐MnO_2_ which consists of a narrow 1 × 1 tunnel structure, shows unfavorable property for the diffusion of Zn^2+^.^[^ ^7^, ^36^ ^]^ However, recent studies by Chen and co‐workers revealed that a reversible insertion/extraction of Zn^2+^ by a phase transition of *β*‐MnO_2_ to form a layered phase Zn‐buserite during initial charge can be achieved via the preaddition of zinc salt with bulky anion (CF_3_SO_3_^−^).^[^ ^31^ ^]^ It is worth to note that the cathode exhibits a high reversible capacity of 225 mAh g^−1^ and excellent cycle ability (94% capacity retention over 2000 cycles). Despite todorokite MnO_2_ consisting of large 3 × 3 tunnels, its tunnels are readily occupied by various cations other than Zn^2+^ and water molecules, which will affect the sites for Zn^2+^ accommodation.^[^ ^54^ ^]^ Hence, todorokite MnO_2_ prepared by hydrothermal treatment of Mg‐buserite only obtained a specific capacity of 98 mAh g^−1^ during the first cycle. It is interesting to note that todorokite MnO_2_ exhibits long‐term cycle ability and good rate performance owing to the large tunnel and the electrostatic shielding effect by the structural water. Unfortunately, the electrochemical mechanism of Zn^2+^ in todorokite MnO_2_ is unclear and still requires a more detailed investigation. Spinel‐type MnO_2_ (*λ*‐MnO_2_) with 3D structure may not be suitable cathode materials for ZIBs due to their limited 3D tunnels.^[^ ^7^, ^14^ ^]^ Later, Yuan et al. demonstrated that preinserted cation *λ*‐MnO_2_ exhibited a specific capacity of 442.6 mAh g^−1^ at 13.8 mA g^−1^, indicating that the Zn^2+^ insertion into *λ*‐MnO_2_ was likely to take place.^[^ ^55^ ^]^ Akhtenskite‐structure (*ε*‐MnO_2_) has also been studied to be a potential cathode material for ZIBs. Akhtenskite‐structure (*ε*‐MnO_2_) is a metastable phase, which exhibits hexagonal symmetry where Mn^4+^ ions are located randomly in one‐half of the face‐shared octahedral sites. For example, Sun et al. reported the interconnected nanoflakes *ε*‐MnO_2_ on carbon fiber paper as binder‐free cathode materials for ZIBs.^[^ ^49^ ^]^ Owing to the microstructure with intensive electrode/electrolyte interface and effective ion diffusion path of the *ε*‐MnO_2_, superior cycling performance of almost 100% retention over 10 000 cycles at 6.5C can be achieved.^[^ ^33^ ^]^ Overall, the previous studies suggest that the large enough tunnel space and layered structure is favorable for allowing the high‐rate insertion/extraction of Zn^2+^. Therefore, the electrochemical properties of MnO_2_ influence significantly on its crystallographic form, which follows the reducing order of *α* = *δ* > *γ* > *λ* > *β*. Owing to the various crystallographic forms, MnO_2_ polymorphs exhibit different insertion thermodynamics and kinetics of H^+^/Zn^2+^. This will further contribute to the different reaction mechanisms in various MnO_2_ polymorphs, leading to different electrochemical properties such as operating voltage, specific capacity, rate capability, and cycling performance of MnO_2_ polymorph for ZIBs.^[^ ^7^, ^14^, ^39^, ^52^, ^53^, ^54^, ^55^ ^]^ Also, it is worth mentioning that the electrolyte composition is another key parameter to determine the electrochemical properties of a Mn‐based cathode. Different anion species electrolyte may significantly influence ion association properties in solution, possible water‐induced side reactions, the stability, and dissolution effect in a Mn‐based cathode.^[^ ^10^, ^14^ ^]^ More research shall be employed to determine the correlation factors between the crystal structures of Mn‐based cathode and electrolyte compositions for optimizing the electrochemical performance for Mn‐based ZIB systems.

#### 3.1.2 Vanadium‐Based Materials

**Figure 3:**
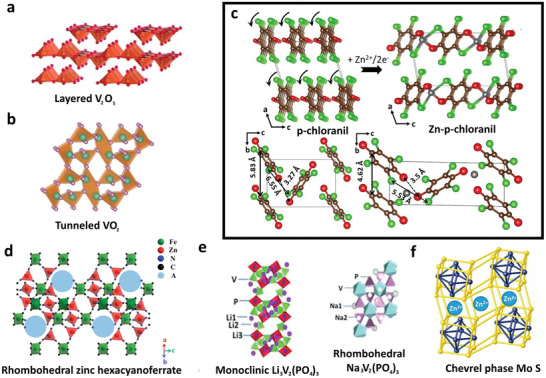
Typical crystal structure cathode materials for ZIBs. a) Layered V_2_O_5_. Reproduced with permission.^[^ ^43^ ^]^ Copyright 2018, American Chemical Society. b) Tunneled VO_2_. Reproduced with permission.^[^ ^11^ ^]^ Copyright 2019, Wiley‐VCH. c) *p*‐chloranil and Zn–*p*‐chloranil obtained by the DFT structural optimization and a squeezing reorientation as the rotation of stacked chloranil columns during the insertion of Zn^2+^ into the *p*‐chloranil molecules. Reproduced with permission.^[^ ^56^ ^]^ Copyright 2018, American Chemical Society. d) Rhombohedral zinc hexacyanoferrate. Reproduced with permission.^[^ ^44^ ^]^ Copyright 2014, Wiley‐VCH. e) NASICON‐structured: monoclinic Li_3_V_2_(PO_4_)_3_ and rhombohedral Na_3_V_2_(PO_4_)_3_. Reproduced with permission.^[^ ^47^ ^]^ Copyright 2016, Springer Nature. f) Chevrel phase Mo_6_S_8_. Reproduced with permission.^[^ ^57^ ^]^ Copyright 2016, American Chemical Society.

Besides manganese‐based materials, vanadium‐based materials especially for vanadium oxides exhibit a large tunnel framework and various oxidation states, showing high potential in Zn^2+^ storage applications. Various structures of vanadium‐based materials, including layered‐ and tunneled‐structure, have been reported as potential cathode materials for ZIBs (see **Figure** **3**a,b). Most of the early stage investigations of vanadium‐based materials for ZIBs are focused on layered vanadium oxides. The common layered‐structure V_2_O_5_ consists of the layer V_4_O_5_ that formed from the sharing edges and corner of VO_5_ square pyramids. It exhibits an interlayer spacing of 0.577 nm, which is much larger than the radius of Zn^2+^ (0.074 nm) and considered as potential cathode materials for ZIBs. Besides, layered V_2_O_5_ has been predicted to deliver a high theoretical capacity of 589 mAh g^−1^, which is ascribed from the two‐electron redox center (V^5+^ → V^3+^) property.^[^ ^43^ ^]^ However, in practice, the layered structure exhibits low capacity and structural instability upon the repeated insertion/extraction of Zn^2+^ ions.^[^ ^58^, ^59^ ^]^ Intensive efforts have been devoted to exploring vanadium oxides with more stable structures to eliminate the capacity fading issues. Besides layered structure V_2_O_5_, tunnel‐type VO_2_ exhibits a reversible Zn^2+^ insertion/extraction performance and superior rate capability in ZIBs.^[^ ^60^ ^]^ This robust structure is comprised of distorted VO_6_ octahedra that share corners and edges with large surface areas, allowing fast diffusion kinetics of Zn^2+^ and structure stability during cycling. Another tunnel‐based material, V_6_O_3_, not only provides a hybrid valence state of V^4+^/V^5+^ but also consists of alternate single and double vanadium oxide layers, providing more accommodation sites for Zn^2+^ storage.^[^ ^61^ ^]^ It is worth noting that a high capacity of 206 mAh g^−1^ can be maintained at 10A g^−1^ after 3000 cycles. Recently, stoichiometric rocksalt vanadium oxynitride with close‐packed face‐centered cubic lattices has been revealed to serve as potential cathode materials for ZIBs.^[^ ^62^ ^]^ The stoichiometric rocksalt vanadium oxynitride was claimed to exhibit a conversion reaction during the first cycle, where high‐valent anion nitrogen (N^3−^) was partially substituted by low‐valent oxygen (O^2−^). This has further resulted in the formation of disordered rocksalt with abundant vacancies/defects, which not only provides intense active sites for zinc ions but also enables rapid diffusion of zinc ions. As a result, the nitrogen‐rich oxynitride cathode shows a high reversible capacity (603 mAh g^−1^, 0.2C) and high rate capability (124 mAh g^−1^ at 600C). In addition, Wan et al. have further reported VOPO_4_, which exhibits a typical layered structure comprising of corner sharing VO_6_ octahedra connected to PO_4_ tetrahedra, as potential cathode materials for high‐performance ZIBs.^[^ ^35^ ^]^ They revealed that the introduction of P—O covalence into the layer of V*_x_*O*_y_* polyhedra can stimulate the occurrence of oxygen redox reaction in the Zn/VOPO_4_ system, enhancing the capacity and average operating voltage of the vanadium‐based ZIBs. Benefiting from the structural variety advantages, manganese‐ and vanadium‐based cathode materials achieve high capacity and high energy density for ZIBs. However, their low intrinsic electronics conductivity, poor capacity retention at low current densities, low structure stability during insertion/extraction, and inevitable material dissolution effects still limited their electrochemical performance for ZIBs.^[^ ^11^ ^]^

#### 3.1.3 Organic‐Based Materials

Recently, organic materials that exhibit weak intermolecular van der Waals forces between molecules and a modest Coulomb repulsion to the diffusing cations, have attracted intensive interests for monovalent and divalent cation energy storage applications.^[^ ^56^ ^]^ Besides, reversible insertion/extraction of divalent cations may take place by molecular reorientation due to their malleable and soft lattice. For example, Kundu et al. reported tetrachloro‐1,4‐benzoquinone with high structural flexibility that exhibits a high capacity of ≥200 mAh g^−1^ with a very small voltage polarization for 1Zn^2+^/2e^−^ reaction (Figure 3c).^[^ ^56^ ^]^ Based on their density‐functional theory (DFT) calculations, a twisted rotation in the molecular column I of *p*‐chloranil is revealed to be useful for accommodating the volume expansion during cycling and stabilizing the electrochemical processes. Zhao et al. also demonstrated the excellent Zn^2+^ storage performance with quinone cathode materials.^[^ ^63^ ^]^ They reported calix[4]quinone that exhibited a stable open bowl structure with eight carbonyls, delivering a high capacity of 335 mAh g^−1^ and a stable cycling performance with a capacity retention of 87% at 500 mA g^−1^ after 1000 cycles. Haupler et Al. have further reported a redox‐active polymer based on a 9,10‐di(1,3‐dithiol‐2‐ylidene)‐9,10‐dihydroanthracene system in combination with a conjugated backbone as cathode material for ZIBs.^[^ ^64^ ^]^ This polymer cathode shows a superior rate capability of up to 120C (30 s) and an ultralong lifetime, of over 10 000 charge/discharge cycles (accompanied by a minor capacity loss of 14%). Although these organic materials show great potential as cathode materials for ZIBs, low solubility in the electrolyte and low electronic conductivity have greatly limited their practical applications. The introduction of a suitable organic‐redox active group with high water stability for a wide range of pH values has been demonstrated to be an alternative approach to promote the zinc storage ability of organic‐based materials. Nonetheless, the research is still in its infancy, which needs further efforts to develop a more effective structural design in organic materials with high electronic conductivity for achieving high specific capacity with long‐term stability.

#### 3.1.4 Prussian Blue Analog Materials

PBA materials that exhibit a face‐centered cubic structure with 3D open framework and large interstitial sites are considered as potential host materials for reversible insertion/extraction of various cations.^[^ ^65^ ^]^ Zhang et al. reported zinc hexacyanoferrates with open framework structure as potential insertion hosts for Zn cations (Figure 3d). An energy density of 100 Wh kg^−1^ is achieved even when the discharge capacity is only 65.4 mAh g^−1^ at 1C due to the high operating voltage.^[^ ^44^ ^]^ However, the PBA‐type cathodes usually suffer from unsatisfactory low capacity, low cycle stability, and inferior rate capability. Recently, Yang et al. have demonstrated that high voltage scanning can effectively activate the reduction of low‐spin Fe(III) coordinated with the C atom of the cyano group in iron hexacyanoferrate (FeHCF) cathodes.^[^ ^66^ ^]^ With a voltage scan at 2.3 V, the Zn–FeHCF hybrid‐ion battery exhibits a gradual increase in capacity. It is worth noting that the unsatisfactory electrochemical performance and low working voltage in FeHCF cathode are mainly caused by the coordination of high spin Fe (Fe atom coordinated with N atom of cyanogroup ) whereas the limited utilization of low‐spin Fe (Fe atom coordinated with C atom of cyanogroup). The increase of capacity is mainly due to the increased contribution from the high discharge voltage plateau at ≈1.5 V, which corresponds to the reduction of low‐spin Fe(III) coordinated with the C atom of cyanogroup. As a result, the activated FeHCF cathode displays significantly enhanced cycling stability with an 82% capacity retention after 5000 cycles and superior rate performance of maintaining 53.2% capacity at a current density of 8 A g^−1^. This work has demonstrated that the controllable chemical and electronics tuning in PBA‐type cathodes by the increasing amounts of redox actives structures may eventually promote their overall electrochemical performance such as cycling stability, rate capability, and operating potential window for ZIBs.

#### 3.1.5 Polyanionic‐Based Materials

Polyanionic compounds with a stable framework, high redox voltage, and vacancies existing in the structure that can store metal ions are also promising candidates for ZIBs.^[^ ^46^, ^47^, ^67^ ^]^ For example, Li et al. synthesized a Na_3_V_2_(PO_4_)_3_ with sodium (Na) super ionic conductor (NASICON) structure as cathode material for ZIBs. Their electrochemical measurements indicate that an ion variation mechanism during the insertion of Zn^2+^ and the Na_3_V_2_(PO_4_)_3_ delivers a reversible capacity of 97 mAh g^−1^ at 0.5C and capacity retention of 74% after 100 cycles.^[^ ^46^ ^]^ Besides, Zhao et al. further used NASICON‐type M_3_V_2_(PO_4_)_3_ such as Li_3_V_2_(PO_4_)_3_ and Na_3_V_2_(PO_4_)_3_ as the cathode materials in a hybrid aqueous electrolyte (Figure 3e).^[^ ^47^ ^]^ They revealed that both Li_3_V_2_(PO_4_)_3_ and Na_3_V_2_(PO_4_)_3_ are relatively stable in aqueous electrolyte and these two phosphate‐based cathode exhibit different electrochemical behaviors. It is worth pointing out that Ko et al. recently reported a different reversible electrochemical behavior of Na_3_V_2_(PO_4_)_3_, which involves a quasi‐two‐stage insertion/extraction of Na^+^ and Zn^2+^, observed for the Zn/Na_3_V_2_(PO_4_)_3_ system. Nevertheless, much more research work is necessary to further improve the understanding of the Zn^2+^ insertion mechanism in the NASICON‐type structure.

#### 3.1.6 Chevrel Phase Materials

Chevrel phase compound with a general formula M*_x_*Mo_6_T_8_, which consists of an octahedral Mo_6_ surrounded by a cubic unit (T_8_) of chalcogenide atoms (Figure 3f), has demonstrated promising potential as cathode materials for ZIBs.^[^ ^57^ ^]^ A 3D network crystal structure creating cavities surrounded by sulfur atoms is formed by the axially bonding between six of the T_8_ to a molybdenum atom of a neighboring cluster. This type of compound exhibits the excellent capability to accommodate various cations with superior ionic and electronic diffusion kinetics. Chae et al. synthesized Mo_6_S_8_ through chemical extraction of copper ions from Cu_2_Mo_6_S_8_.^[^ ^68^ ^]^ They revealed that the Zn^2+^ insertion/extraction into Mo_6_S_8_ occurred stepwise and the charge/discharge process for the Mo_6_S_8_ over ZnMo_6_S_8_ to Zn_2_Mo_6_S_8_ were reversible. Nonetheless, the insertion voltage was too low (0.35 V), resulting in low energy density and an unsuitable cathode candidate for ZIBs.

In summary, various cathode materials with unique structure features such as tunnel‐based, layered‐based, and 3D framework‐based structures have been investigated as cathode materials for ZIBs.^[^ ^7^, ^14^, ^39^, ^52^, ^53^, ^54^, ^55^ ^]^ Generally, there is a strong relationship between the types of cathode materials and Zn^2+^ storage abilities, that modulates the overall energy storage reaction mechanism such as operating potential platforms and reaction mechanisms (see **Table** **1**). It is worth noting that the unique features of their crystal structure also play important role in determining their zinc storage ability. For example, various electrochemical reaction mechanisms have been reported for various MnO_2_ polymorphs.^[^ ^10^, ^11^, ^14^ ^]^ More efforts shall be focused on the more precise investigation of polyhedral or crystal structures for the Zn^2+^ storage reaction process by more advanced characterization to explore other potential cathode materials with zinc‐intercalation favorable structures and morphologies for ZIBs.

**Table 1:** Comparison of the various typical cathode materials that are widely investigated for ZIBs

Typical cathode materials	Structure benefits	Operation voltage platforms [V]	Reaction mechanisms	Limitations
Mn‐based material^[^ ^7^, ^9^, ^10^, ^14^, ^39^, ^52^, ^53^, ^54^, ^55^ ^]^	Polymorphs with various tunnel, layered structure, multivalence state	1.3–1.4	Reversible Zn^2+^ insertion/extraction, coinsertion of Zn^2+^ and H^+^ mechanism, conversion reaction mechanism	Poor electrical conductivity, Mn^2+^ dissolution, structural instability during cycling, insertion mechanisms, performance optimization of cathode materials
V‐based material^[^ ^9^, ^10^, ^11^ ^]^	Various structures such as layered‐ and tunneled structure and various oxidation states	0.9–1.1	Reversible Zn^2+^ insertion/extraction, oxygen redox reaction	Low intrinsic electronic conductivity, poor capacity retention at low current densities, low structure stability during insertion/extraction, and inevitable material dissolution effects
Organic‐based material^[^ ^56^, ^63^, ^64^ ^]^	Weak intermolecular van der Waals forces between molecules and a modest Coulomb repulsion, malleable and soft lattice	1.0–1.1	Reversible Zn^2+^ insertion/extraction	Low solubility in electrolyte and low electronic conductivity
Prussian Blue Analog materials^[^ ^44^, ^65^ ^]^	3D open framework and large interstitial sites	1.3–1.7	Reversible Zn^2+^ insertion/extraction	Unsatisfactory low capacity, low cycle stability, and inferior rate capability
Polyanionic‐based materials^[^ ^46^, ^47^, ^67^ ^]^	Stable framework with NASICON structure, vacancies existing in the structure that can store metal ion	1.6–1.7	Reversible Zn^2+^ insertion/extraction	Electrochemical performance is highly affected by the electrolyte composition
Chevrel phase materials^[^ ^57^, ^68^ ^]^	3D network crystal structure creating cavities surrounded by sulfur atoms	0.5–0.6	Reversible Zn^2+^ insertion/extraction	Insertion voltage is too low, low capacity

### 3.2 Crystal Defect Engineering

Crystal defect engineering, which provides optimization of electrochemical active surface area for effective electrochemical reaction, is an effective approach to tackle the current limitations of cathode materials for ZIBs.^[^ ^17^, ^18^, ^19^, ^69^, ^70^ ^]^

**Figure 4:**
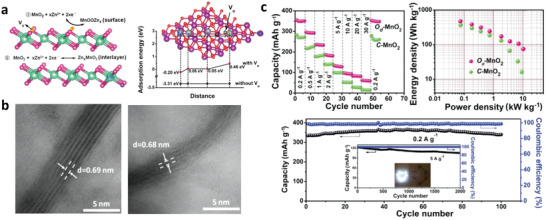
a) Scheme of oxygen‐deficient *σ*‐MnO_2_ for Zn^2+^ storage and theoretical calculated surface adsorption energies for Zn^2+^ of defect‐free and oxygen‐deficient *σ*‐MnO_2_. b) High resolution annular bright‐field scanning transmission electron microscopy (ABF‐STEM) images of defect‐free and oxygen‐deficient *σ*‐MnO_2_. Irregular lattices resulting from shrinkage of the interlayer spacing and the fragmented layer are observed in oxygen‐deficient *σ*‐MnO_2_, which attributed to high density of oxygen vacancies in oxygen‐deficient *σ*‐MnO_2_. c) Rate performance, the Ragone plots, and cycling performance of defectless and oxygen‐deficient *σ*‐MnO_2_. Reproduced with permission.^[^ ^18^ ^]^ Copyright 2019, Wiley‐VCH.

Generally, manganese oxides exhibit a low utility rate of surface active sites, significantly impeding their electrochemical performance for ZIBs. Introducing defects such as oxygen vacancy and cation deficiency in manganese oxide cathode materials can lower Gibbs free energy of Zn^2+^ adsorption, facilitating the insertion/extraction of Zn^2+^. For example, Xiong et al. designed an oxygen‐deficient MnO_2_ as cathode material for ZIBs.^[^ ^18^ ^]^ They revealed that the existence of oxygen vacancies in the MnO_2_ enabled an enhanced electrochemical performance owing to the high accessible electrochemical active surface area and the generation of available electrons by the formation of Zn—O bonding (see **Figure** **4**a,b). As a result, the oxygen‐deficient manganese oxide exhibits a high capacity of 345 mAh g^−1^, superior rate capability, and long‐term cycling stability at a low current density of 0.2 A g^−1^ (see Figure 4c). The recent theoretical study by Han et al. also demonstrated that the introduction of oxygen defect in the *β*‐MnO_2_ with a relatively narrow tunneled pathway could effectively facilitate the insertion of H^+^ into *β*‐MnO_2_.^[^ ^69^ ^]^ They further confirmed their theoretical conjecture by assembling aqueous Zn/*β*‐MnO_2_ battery. A high capacity of 302 mAh g^−1^ and superior cycling performance with a capacity retention of 94% after 300 cycles are achieved with the oxygen‐deficient *β*‐MnO_2_ cathode. Liao et al. further revealed that oxygen‐deficient vanadium oxide cathode exhibited unprecedented stability over 200 cycles with a superior capacity of 400 mAh g^−1^, reaching 95% utilization of its theoretical capacity.^[^ ^71^ ^]^ They suggested that the introduction of oxygen vacancies in vanadium oxide by synthetic strategy favored divalent Zn^2+^ reaction kinetics and enhanced Zn^2+^ reaction pathway for high reversible Zn^2+^ insertion/extraction. Besides, Li et al. have further investigated the effects of oxygen vacancies on Zn^2+^ intercalation in VO_2_ for aqueous ZIBs.^[^ ^72^ ^]^ They have revealed that the generation of oxygen vacancies in VO_2_ cathode can result in a larger tunnel structure along the *b*‐axis, which enhances the reactive kinetics and promotes Zn^2+^ storage capability in VO_2_ cathode. As a consequence, a high specific capacity of 375 mAh g^−1^ at a current density of 100 A g^−1^ and excellent long‐term cycling stability with retained specific capacity of 175 mAh g^−1^ at 5 A g^−1^ over 2000 cycles were achieved.

**Figure 5:**
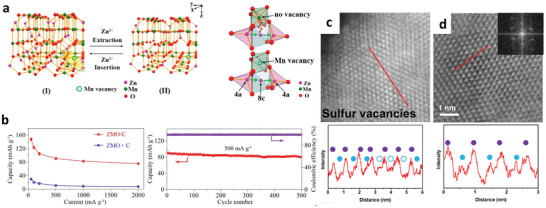
a) Illustration of the insertion/extraction of Zn^2+^ into zinc manganese oxide spinel structure and proposed diffusion pathway of Zn^2+^ into the spinel‐structured zinc manganese oxide without and with Mn deficiency. b) Rate capability and cycling performance of pristine and Mn‐deficient zinc manganese oxide. Reproduced with permission.^[^ ^74^ ^]^ Copyright 2016, American Chemical Society. c,d) Comparison of high magnification high‐angle annular dark‐field imaging (HAADF) STEM images with line profile plot of defect‐rich and defect‐free MoS_2_ nanosheets. The intensity profile across the red line in (c) with a sudden drop intensity on the sulfur peaks indicates the presence of sulfur vacancies. Reproduced with permission.^[^ ^17^ ^]^ Copyright 2018, Elsevier.

Other spinel structures such as ZnMn_2_O_4_ and MnMn_2_O_4_(Mn_3_O_4_) may not be suitable cathode materials for ZIBs due to their limited 3D tunnel. Also, owing to the high electrostatic interaction between Zn^2+^ and the lattice of spinel structures such as ZnMn_2_O_4_, the Zn^2+^ insertion/extraction becomes more difficult.^[^ ^73^ ^]^ The cation‐deficient spinel ZnMn_2_O_4_ was then prepared to lower the electrostatic barrier and promote the Zn^2+^ diffusion in the host.^[^ ^73^ ^]^ A new mechanism with the insertion and extraction of Zn^2+^ into the Zn—O tetrahedron sites of zinc manganese oxide spinel is illustrated in **Figure** **5**a; therefore, the basic spinel lattice can be maintained during the cycling process. They further suggested that the presence of abundant Mn vacancies in zinc manganese oxide is beneficial for the Zn^2+^ diffusion. It is worth noting that capacity retention of 94% over 500 cycles at 500 mA g^−1^ can be achieved with the cation‐deficient spinel ZnMn_2_O_4_ (see Figure 5b). Overall, this work has revealed that the cation defects in ZnMn_2_O_4_ are beneficial to modulate the electronic conductivity, Zn^2+^ transport kinetics, and the energy barrier of Zn^2+^ mobility.

Besides, Zhu et al. developed an in situ electrochemical approach for the activation of MnO by the introduction of Mn cation defects.^[^ ^19^ ^]^ They proposed the generated Mn defect via a charging process, which can transform the electrochemically inactive MnO into a highly active cathode for aqueous ZIBs. It is important to note that the proposed cathode exhibited a reversible Zn^2+^ insertion/extraction mechanism without structural collapse during cycling. Apart from the oxide‐based materials, defect engineering in layered metal chalcogenides with an improved electrochemical performance for ZIBs has also been reported. Generally, 2H‐MoS_2_ exhibits poor electrochemical activities toward Zn^2+^.^[^ ^28^ ^]^ Recently, Xu et al. reported sulfur‐deficient MoS*_x_* nanosheets as cathode for ZIBs, which delivered a high reversible capacity as compared to the defect‐free MoS_2_.^[^ ^17^ ^]^ The defect‐rich MoS_2_ consists of numerous edge sites and vacancies, which provide accommodation sites for Zn^2+^ (see Figure 5c,d), converting poor Zn^2+^ interaction host materials into functional active cathode materials for ZIBs and thus resulting in a high reversible capacity.

The electrochemical process generally involves three primary processes, namely, mass diffusion, electron transfer, and cathode/electrolyte reaction. The overall performance of a cathode is usually significantly influenced by the charge transport properties and redox‐active site capabilities. Defect engineering has been a promising approach for tuning the electrical structure and surface morphology, resulting in novel physicochemical properties or strong synergistic effects that potentially improved electrochemical performance. Yet fundamentally, most of the reported works simply propose that the existence of defects has a powerful effect on electrochemical performance. Often other aspects such as controllable defect types and concentrations which may alter the electrochemical performance of cathode material were not taken into consideration. Particular attention should be employed to evaluate the defect‐drive electrochemical performance via precise synthesis.

### 3.3 Interlayer Engineering

Owing to the (de)intercalation‐type storage mechanism of ZIBs, the hydrated Zn^2+^ generally prefers to store in the layered structure. Interlayer engineering is another method to optimize microstructures, which plays an important role in endowing the electrode materials with high Zn^2+^ storage performance. Interlayer engineering can significantly enhance the diffusion kinetics of ions with large sizes and multivalence via reducing their diffusion barriers.^[^ ^27^ ^]^ Besides, interlayer engineering also endows the cathode materials with a large specific surface area, which improves their electrochemical performance.^[^ ^22^, ^75^ ^]^

**Figure 6:**
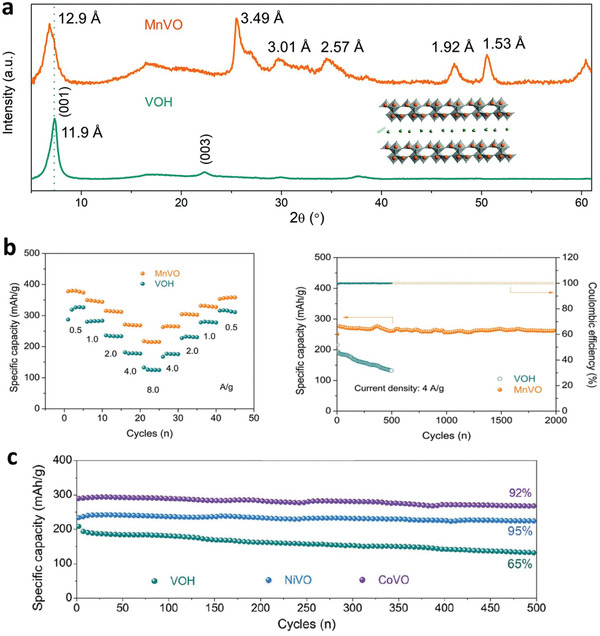
a) XRD patterns of Mn‐expanded hydrated vanadium pentoxide (MnVO) and hydrated vanadium pentoxide (VOH). b) Comparison of rate capability and cycle stability of MnVO and VOH. c) Cycling stability measured at 4 A g^−1^ of Ni‐expanded, Coexpanded, and pristine hydrated vanadium pentoxides. Reproduced with permission.^[^ ^76^ ^]^ Copyright 2019, The Royal Society of Chemistry.

Various efforts are developed to insert foreign species into the host layered lattice.^[^ ^21^, ^22^, ^27^, ^76^ ^]^ For example, metal‐ion intercalation, including Li^+^, Na^+^, K^+^, Mn^2+^, and Zn^2+^, is explored to improve the performance of V_2_O_5_ cathode materials.^[^ ^77^, ^78^, ^79^, ^80^, ^81^ ^]^ It is worth to note that a recent report by Liu et al. revealed that the chemical insertion of Mn(II) cations in hydrated vanadates can act as structural pillars, which expand the interplanar spacing from 11.9 to 12.9 Å, linking the adjacent layers and partially reducing the pentavalent vanadium cations to tetravalent (see **Figure** **6**a).^[^ ^76^ ^]^ As a result, manganese‐expanded hydrated vanadate achieved a high specific capacity of 415 mAh g^−1^ at 50 mA g^−1^ and an excellent cycling performance with a capacity retention of 92% over 2000 cycles at 4 A g^−1^ (see Figure 6b). They further demonstrated improved Zn^2+^ storage performance in expanded hydrated vanadates by introducing other intercalated transition metal cations such as Ni and Co (see Figure 6c). Nonetheless, the specific capacity of metal vanadate cathode materials is still limited by the large molecular weight and volume of metal ions. In addition, the metal‐ion intercalated host materials still suffer from structural damage owing to the insertion of metal ions between layers.

**Figure 7:**
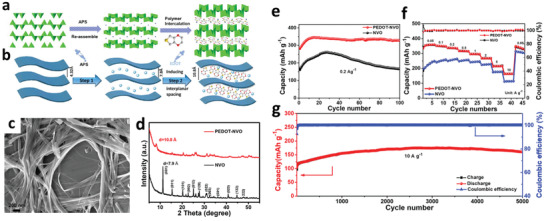
a,b) Illustration of structural changes and description of simulation changes during the preparation process of PEDOT‐intercalation NVO‐layered material. c) Scanning electron microscope (SEM) images of PEDOT‐intercalation NVO‐layered material. d) XRD patterns of PEDOT–NVO and NVO samples. e,f) Cycling performance at low current density of 0.2 A g^−1^ and rate performance of PEDOT–NVO and NVO samples. g) Cycling performance at high current density of 10 A g^−1^ of PEDOT–NVO sample. Reproduced with permission.^[^ ^83^ ^]^ Copyright 2020, Elsevier.

Other intercalating agents such as conductive polymers have also been investigated to modulate the interlayer spacing for zinc cathode materials. For example, Huang et al. reported mesoporous structure polyaniline inserted into MnO_2_ nanolayers by an interface reaction, which eliminated phase transformation and delivered a stable cycling performance for ZIBs.^[^ ^82^ ^]^ Besides, Bin et al. synthesized the redox intercalative polymerization of 3,4‐ethylenedioxythiophene into ammonium vanadate oxide (PEDOT–NVO) via a facile synthesis method, as illustrated in **Figure** **7**a–c.^[^ ^83^ ^]^ The obtained PEDOT‐intercalated NVO exhibits an expanded interplanar spacing crystal lattice (Figure 7d), which leads to an enhancement in the diffusion of cation species intercalating into the crystal lattice of the NVO. As a result, the hybrid of organic–inorganic‐intercalated NVO layer cathode exhibits a high capacity with good cycling stability and excellent rate capability (Figure 7e–g). As mentioned earlier, MoS_2_ is an intrinsically inactive Zn^2+^ insertion/extraction host material to store Zn^2+^. Liang et al. reported an effective approach by tuning the interlayer spacing and hydrophilicity of MoS_2_ via oxygen incorporation and thus converted intrinsically inactive Zn^2+^ insertion/extraction hosts into efficient Zn^2+^ storage materials.^[^ ^27^ ^]^ Consequently, the capacity of the activated MoS_2_ was enhanced by 10 times as compared to the inactive MoS_2_, delivering a high capacity of 232 mAh g^−1^ at 100 mA g^−1^. Zhi and co‐workers further demonstrated the excellent Zn^2+^ storage performance with interlayer‐spacing‐expanded MoS_2_ nanosheets (*ε*‐MoS_2_) on carbon fiber.^[^ ^22^ ^]^ The expanded interlayer spacing of *ε*‐MoS_2_ nanosheets enabled effective ion diffusion kinetics and low energy barrier for Zn^2+^ insertion/extraction, resulting in a good specific capacity of 202.6 mAh g^−1^ at 100 mA g^−1^, a high energy density of 148.2 Wh kg^−1^ and high cyclic stability.

In short, interlayer engineering of layered materials plays a critical role in tuning properties and improving the performance of cathode materials for ZIBs. Nonetheless, challenges still exist in synthesizing microstructures with controllable amounts of intercalated foreign species, interlayer distance, and composition via large‐scale and cost‐effective synthesis process. Well‐developed guidelines on the selection of suitable intercalated species for obtaining optimal interlayer spacing and electrochemical performance are thus required.

### 3.4 Morphological Design

#### 3.4.1 Low‐Dimensional Structures

**Figure 8:**
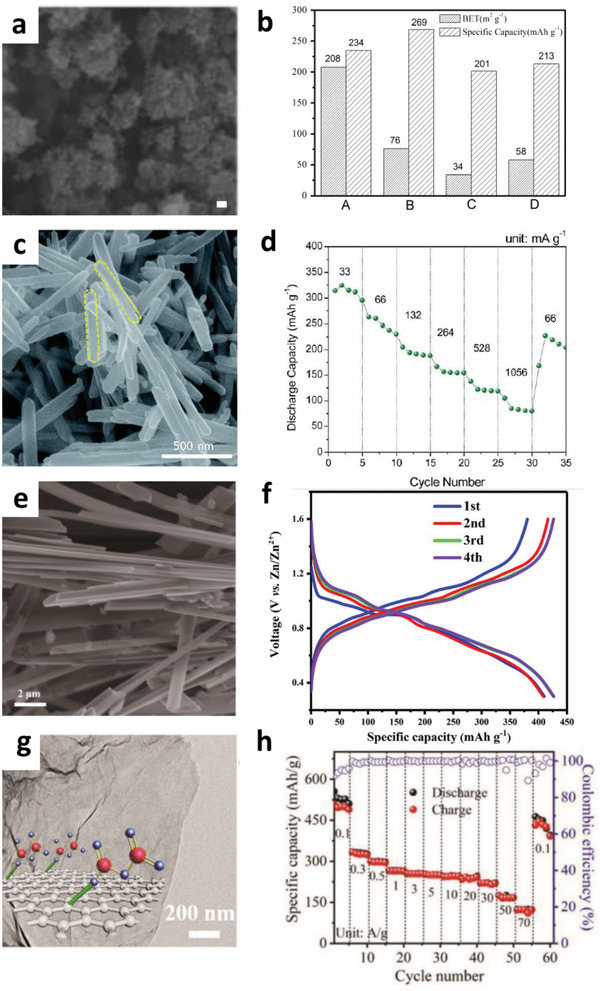
a,b) SEM image of *α*‐MnO_2_ nanoparticles and comparison of the first discharge capacity and Brunauer–Emmett–Teller (BET) measurements for *α*‐MnO_2_ with other MnO_2_ structures for ZIBs, where A is *α*‐MnO_2_ aggregated spherical nanoparticles and cylindrical nanorods, B is *δ*‐MnO_2_ nanorods, C is interlocked short nanosheets *γ*‐MnO_2_, and D is aggregated nanorods *β*‐MnO_2_. Reproduced with permission.^[^ ^36^ ^]^ Copyright 2012, Elsevier. c,d) SEM image and rate performance of *β*‐MnO_2_ nanorod cathode for ZIBs. Reproduced with permission.^[^ ^53^ ^]^ Copyright 2017, The Royal Society of Chemistry. e,f) SEM image and discharge–charge profiles in the first four cycles at a current density of 0.1 A g^−1^ of CuV_2_O_6_ nanobelt cathode for ZIBs. Reproduced with permission.^[^ ^84^ ^]^ Copyright 2019, American Chemical Society. g,h) SEM image, rate performance, and Coulombic efficiency of A‐V_2_O_5_/G cathode for ZIBs. Reproduced with permission.^[^ ^103^ ^]^ Copyright 2020, Wiley‐VCH.

Generally, low‐dimensional materials demonstrate excellent mechanical flexibility, high specific surface area, numerous active sites, and high chemical stability than their bulk counterparts. Their superior structural features have attracted intensive attention to the design of low‐dimensional microstructures for high‐performance electrode materials. Growth of 0D nanocrystals has been widely developed to promote reaction kinetics and accommodate the volume expansion of the electrode materials during the insertion/extraction process. For example, Wei et al. synthesized various manganese dioxides with different tunnel structures and morphologies via a common liquid coprecipitation method based on the redox reaction of Mn^4+^ and Mn^2+^ for ZIBs.^[^ ^36^ ^]^ Spherical nanoparticles of 20 nm in diameter, nanorods of 40–100 nm in length and 20 nm in diameter, or interlocked nanosheet of 10–20 nm thickness can be obtained based on the preparation procedure. Owing to the low‐dimensional features of high surface area and abundant active sites for the Zn^2+^ storage, the manganese dioxide composed of spherical nanoparticles and cylindrical nanorods exhibited a high specific surface area of 208 m^2^ g^−1^ and achieved a high first charge/discharge capacity of 234 mAh g^−1^ (**Figure** **8**a,b).

1D microstructures, including nanowires,^[^ ^85^, ^86^, ^87^, ^88^, ^89^ ^]^ nanorods,^[^ ^53^ ^]^ and nanobelts^[^ ^84^, ^90^, ^91^ ^]^ have attracted intensive attention for energy storage applications due to their distinct properties, such as large surface‐to‐volume ratio and inhibition to agglomeration. For example, Islam et al. demonstrated that *β*‐MnO_2_ nanorods with exposed (101) planes can be a potential cathode material for ZIBs (Figure 8c).^[^ ^53^ ^]^ *β*‐MnO_2_ nanorods with exposed (101) planes were synthesized by a rapid microwave‐assisted hydrothermal reaction in 10 min. They revealed that *β*‐MnO_2_ nanorods exhibited a higher discharge capacity of 270 mAh g^−1^ at 100 mA g^−1^ and excellent rate capability as compared to bulk *β*‐MnO_2_, demonstrating that the unique rod‐shaped materials enhance the insertion/extraction of Zn^2+^ into/from the nanorod structures (Figure 8d). Besides, CuV_2_O_6_ nanobelts have been synthesized by a facile hydrothermal method (Figure 8e).^[^ ^84^ ^]^ Owing to the rational designed morphology of CuV_2_O_6_, enhanced Zn^2+^ diffusion rate and better electrochemical performance with a high reversible capacity of 427 mAh g^−1^ at a current density of 0.1 A g^−1^, and superior stability with a minor capacity loss of 0.7% after 3000 cycles at 5 A g^−1^ can be achieved (Figure 8f). They further revealed that the Zn^2+^ storage performance of the 1D microstructure can be further enhanced by combining with carbonaceous materials. In their work, they demonstrated a significantly improved specific capacity by at least 30% increment.

Besides 1D microstructures, 2D microstructures with an atomically thin layer structure, including layered metal oxides,^[^ ^20^, ^26^, ^92^, ^93^, ^94^, ^95^, ^96^, ^97^, ^98^, ^99^, ^100^, ^101^ ^]^ metal chalcogenides,^[^ ^22^, ^27^ ^]^ and MXene^[^ ^23^ ^]^ have been investigated for Zn^2+^ storage. Owing to the layer structure with large interlayer spacing and high conductivity, transition metal dichalcogenide nanosheets such as MoS_2_ and VS_2_, demonstrate great potential for the insertion/extraction of multivalent ions such as Zn^2+^.^[^ ^23^, ^97^, ^102^ ^]^ For example, Pan and co‐workers synthesized VS_2_ nanosheets via a facile hydrothermal process.^[^ ^102^ ^]^ When employed as the cathode material for ZIBs, VS_2_ nanosheets delivered high capacity and exhibited long‐term cyclic stability owing to the large interlayer spacing of 5.76 Å that enabled the insertion/extraction of Zn^2+^ (0.74 Å) and high conductivity of VS_2_ nanosheets.

Recently, Wang et al. developed a novel 2D heterostructure of ultrathin amorphous vanadium pentoxide uniformly grown on graphene (A‐V_2_O_5_/G) by 2D template based on ion‐adsorption approach as cathode material for ZIBs (Figure 8g).^[^ ^103^ ^]^ The 2D sandwich‐like composite consists of ultrathin nanosheet (≈ 5 nm) with short ion diffusion, numerous active sites, and high electrical conductivity, which significantly promote the prominent capacitive‐controlled kinetics process as well as facilitate the Zn^2+^ intercalation reaction. As a result, the 2D A‐V_2_O_5_/G heterostructures delivered a high specific capacity of 489 mAh g^−1^ and an outstanding rate capability of 123 mAh g^−1^ even at 70 A g^−1^ (Figure 8h). Zhao et al. designed a robust structure of nanosheet MnO_2_H_0.16_(H_2_O)_0.27_ (MON) with a thickness of ≈2.5 nm as a promising cathode material for ZIBs.^[^ ^97^ ^]^ High electrochemical performance of the Zn/MON cell with a high energy density of 228.5 Wh kg^−1^ and superior cycling stability (capacity retention of 96% at 0.5C after 300 cycles) was achieved, owing to the ultrathin layer structure combined with H^+^/Zn^2+^ synergistic insertion/extraction mechanism.

#### 3.4.2 Hierarchical Structures

With the combined advantages of the low‐dimensional microstructures and large 3D framework, hierarchical structures show great potential for Zn^2+^ storage. More specifically, low‐dimensional units can facilitate the diffusion of Zn^2+^/electrons and provide large electrode/electrolyte active contact surface area, while large 3D frameworks can provide stability of the structure with effective buffering on the volume change during charge/discharge process.

**Figure 9:**
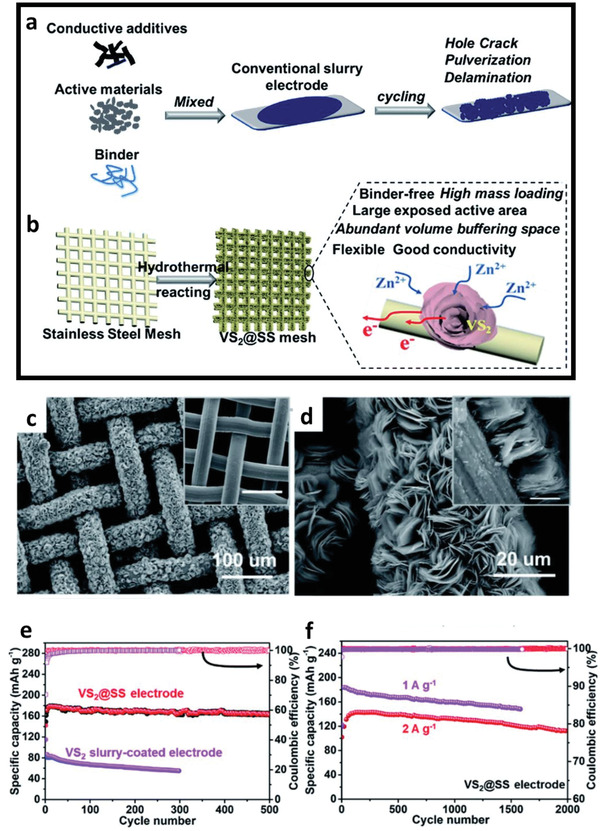
Schematic illustration of the preparation processes for a) the conventional slurry‐coated electrode, and b) the binder‐free hierarchical VS_2_ grown on a stainless‐steel mesh substrate (VS_2_@SS) electrode. c,d) SEM images of VS_2_@SS under different magnifications and the inset in (d) is the cross‐section image of VS_2_@SS. e,f) Cycling performance of VS_2_@SS electrode and VS_2_ slurry‐coated electrode at 0.5 and 2 A g^−1^. Reproduced with permission.^[^ ^104^ ^]^ Copyright 2019, The Royal Society of Chemistry.

Recently, various hierarchical structures have been synthesized for ZIB applications.^[^ ^22^, ^103^, ^104^, ^105^, ^106^, ^107^, ^108^, ^109^, ^110^ ^]^ For example, the direct synthesis of binder‐free hierarchical 1T VS_2_ flower‐like structure on stainless steel mesh by hydrothermal method has been reported by Jiao et al. for ZIBs.^[^ ^104^ ^]^ The synthesis of freestanding materials on conductive substrates which can be directly used as cathode materials for energy storage applications enables higher gravimetric/volumetric energy density as compared to the conventional slurry‐coated electrodes which consist of inactive materials such as the binder and conductive additives (see **Figure** **9**a,b). The open‐structure 1T phase VS_2_ flowers (Figure 9c,d) demonstrated a high reversible capacity of 198 mAh g^−1^ and stable cycling performance with 80% capacity retention over 2000 cycles at 2 A g^−1^ (Figure 9e,f).

In addition, the hierarchical structure which comprises of active materials and highly conductive 3D carbonaceous networks (e.g., graphene and carbon nanotubes (CNTs)), have been synthesized to improve the Zn^2+^ storage performance. For example, Xu et al. prepared a freestanding and highly porous composite consisting of bilayer Na*_x_*V_2_O_5_·*n*H_2_O nanobelts, carbon nanotubes, and reduced graphene oxide (RGO) with 3D interconnected structure.^[^ ^106^ ^]^ With a unique hierarchical nanoarchitecture of the combined structure between 1D and 3D scaffold, the freestanding composites delivered a capacity of 459.1 mAh g^−1^ at 0.5 A g^−1^, superior rate capability, and excellent cycling stability of 83.1% retention after 1800 cycles at 10 A g^−1^. A novel hydrophilic carbon substrate by acid‐treated natural halloysite and carbon nanotube for loading V_3_S_4_ have been reported by Liu et al.^[^ ^107^ ^]^ The coupling electrode materials with carbon substrate exhibited a high specific capacity of 148 mAh g^−1^ at 0.5 A g^−1^ and a high energy density of 155.7 Wh kg^−1^ owing to the combined advantage of the high conductivity and the formation of the hydrophilic surface/interface of the composite electrode.

Fabrication of hierarchical structures that consist of a hybrid structure of layered materials with different carbon materials, has been reported to be an effective approach to promote the electrochemical performance of 2D microstructures.^[^ ^26^, ^92^, ^94^ ^]^ For example, Huang and co‐workers reported a MnO_2_ nanosheet/RGO composite structure.^[^ ^91^ ^]^ The RGO channels with mesopores not only increased the conductivity but also accommodated the volume change of MnO_2_ during the charge/discharge process, resulting in a significantly enhanced capacity (332.2 mAh g^−1^ at 0.3 A g^−1^), improved rate capability, and cyclability. Zhi and co‐workers reported expanded interlayer spacing MoS_2_ nanosheets on a carbon cloth by a one‐step glucose‐assisted hydrothermal approach.^[^ ^22^ ^]^ The as‐synthesized composites demonstrated a high specific capacity of 206 mAh g^−1^ at 100 mA g^−1^, an energy density of 148.2 Wh kg^−1^, and superior cycling performance with a capacity retention of 98.6% after 600 cycles. Besides, Wu et al. synthesized MnO_2_‐nanosheet‐assembled hollow polyhedron on carbon cloth (CC) via a rapid hydrothermal method using zeolitic imidazole framework (ZIF)‐67 as self‐sacrificing template.^[^ ^26^ ^]^ The hollow MnO_2_ nanosheet polyhedron provided a large surface area which facilitated the penetration of electrolyte and accommodated volume change during charge and discharge processes, whereas the carbon cloth substrate promoted the electron transportation. Benefiting from the combined structural advantages, the MnO_2_/CC delivered a high reversible capacity of 263.9 mAh g^−1^ at 1.0 A g^−1^ after 300 cycles, which was much better than that of a commercial MnO_2_ electrode.

#### 3.4.3 Hollow Structures

Owing to the well‐defined shell and interior voids, effective diffusion path for ions and electrons, and large active surface area, hollow structured nanomaterials have shown the potential to be excellent electrode materials for ZIBs. In brief, the interior hollow architecture accommodates the volume expansion during the charge/discharge process, enabling excellent cycling stability. Meanwhile, most of the hollow structure can retain their original structure even after long cycling. Besides, the large surface area enables the hollow structures with intensive electrochemical active sites and the large effective contact area between electrolyte and electrode for efficient ion and electron diffusion, resulting in improved reversible capacity. Many previous works have reported that the energy storage ability of hollow structure materials can be further optimized via the design of the external shape, shell architecture, internal configuration, size, and chemical composition.^[^ ^26^, ^111^, ^112^, ^113^, ^114^, ^115^, ^116^ ^]^

Numerous hollow structures have been employed as cathode materials for sodium‐ion batteries (SIBs) and LIBs and showed excellent electrochemical properties.^[^ ^119^, ^120^, ^121^, ^122^ ^]^ Nonetheless, hollow structuring in cathode materials for ZIBs is less reported. Recently, hollow spherical structures have been investigated as electrode materials for ZIBs.^[^ ^25^, ^115^, ^118^, ^123^ ^]^ For example, Chen et al. synthesized hollow V_2_O_5_ nanospheres with a shell thickness of 50 nm and a diameter of 450 nm through a template‐free solvothermal method followed by calcination treatment.^[^ ^25^ ^]^ The nanosized hollow structure buffered the structural volume change, provided efficient ion/electron diffusion paths, and enhanced the surface capacitive behavior, showing an ultrahigh reversible capacity of 327 mAh g^−1^ at 0.1 A g^−1^, excellent rate capability, and cycling stability with 147 mAh g^−1^ after 6000 cycles at 10 A g^−1^. Even at a high current density of 15 A g^−1^, a high capacity of 122 mAh g^−1^ can still be maintained after 10 000 cycles.

**Figure 10:**
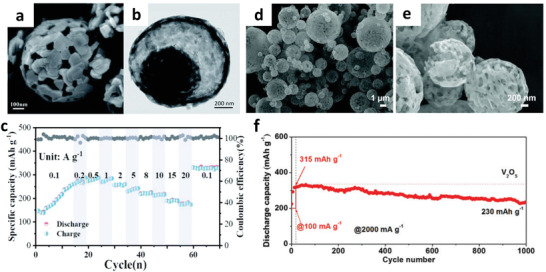
a,b) SEM and TEM image of the V_2_O_5_–YS microspheres. c) Rate performance of the V_2_O_5_–Yolk‐Shell (YS) microspheres. Reproduced with permission.^[^ ^117^ ^]^ Copyright 2020, The Royal Society of Chemistry. d,e) SEM images of porous V_2_O_5_ microspheres. f) Cycling performance of porous V_2_O_5_ microspheres at a current density of 2 A g^−1^. Reproduced with permission.^[^ ^118^ ^]^ Copyright 2019, The Royal Society of Chemistry.

Besides, Li et al. reported an anhydrous V_2_O_5_ yolk–shell cathode synthesized by a facile template‐free solvothermal process (**Figure** **10**a,b).^[^ ^117^ ^]^ Owing to the yolk–shell architectures, the obtained composites delivered a high reversible capacity of 410 mAh g^−1^ at 100 mA g^−1^ and a stable cycling performance with a capacity retention of 80% over 1000 cycles at 5 A g^−1^ (Figure 10c). Hu et al. reported porous V_2_O_5_ microspheres via the spray‐drying approach as cathode materials for ZIBs (Figure 10d,e).^[^ ^118^ ^]^ They suggested that the porous microspheres offered a structural advantage on accommodating volume expansion during cycling and provided effective ion diffusion pathway. As a result, the porous V_2_O_5_ microspheres demonstrated an excellent discharge capacity of 401 mAh g^−1^ at 100 mA g^−1^, superior rate capability (234 mAh g^−1^ at 5000 mA g^−1^), and cycling performance with a capacity retention of 73% over 1000 cycles (Figure 10f).

Overall, morphology engineering enables the construction of cathode microstructure materials with morphology‐dependent benefits such as high surface to mass/volume ratio, improved conductivity, intensive chemical active sites, and stable framework that can accommodate the volume expansion during the cycling process. It can be concluded that morphology engineering improves the structure stability, enhances the overall reaction kinetic, and enables high specific capacity without altering the overall redox reaction of cathode material in ZIBs (see **Table** **2**). However, most of these achievements were obtained with relatively low areal loading of cathode material (<1 mg cm^−2^) which is much far from the practical application standard for ZIBs.^[^ ^124^ ^]^ To achieve practical application of ZIBs, the development of advanced self‐standing cathodes with an industrial‐level areal capacity (≈35 mAh cm^−2^), superior gravimetric capacity, excellent rate, and cycling stability simultaneously is essential.

**Table 2:** Summary of electrochemical performance of various cathode materials with a diverse of morphologies in aqueous ZIBs

Cathode material	Morphology	Reaction mechanism	Potential window [V]	Main anodic and cathodic peaks [V]	Discharge capacity
*α*‐MnO_2_^[^ ^36^ ^]^	Nanoparticles (20 nm)	Zn^2+^ ion insertion/extraction	1–2	1.75 and 1.25	234 mA h g^−1^ (100 mA g^−1^)
*δ*‐MnO_2_^[^ ^36^ ^]^	Nanoplatelets (10–20 nm)	Zn^2+^ ion insertion/extraction	1–2	1.7 and 1.3	213 mA h g^−1^ (100 mA g^−1^)
*γ*‐MnO_2_^[^ ^36^ ^]^	Spherical agglomerate nanosheets	Zn^2+^ ion insertion/extraction	1–2	1.7 and 1.35	269 mA h g^−1^ (100 mA g^−1^)
*β*‐MnO_2_^[^ ^36^ ^]^	Spherical agglomerate nanosheets	Zn^2+^ ion insertion/extraction	1–2	1.7 and 1.3	201 mA h g^−1^ (100 mA g^−1^)
*β*‐MnO_2_^[^ ^53^ ^]^	Nanorods	Zn^2+^ ion insertion/extraction	1–1.8	1.65/1.6 and 1.38/1.1	270 mA h g^−1^ (100 mA g^−1^)
CuV_2_O_6_^[^ ^80^ ^]^	Nanobelts	Reduction and oxidation of Cu^2+^, Zn^2+^ ion insertion/extraction	0.3–1.6	1.13/1.01/0.85 and 1.06/0.88/0.76	361 mA h g^−1^ (200 mA g^−1^)
VS_2_^[^ ^98^ ^]^	Nanosheet	Zn^2+^ ion insertion/extraction	0.4–1.0	0.72/0.75 and 0.58/0.63	159.1 mA h g^−1^ (100 mA g^−1^)
V_2_O_5_/graphene^[^ ^103^ ^]^	Ultrathin layer 2D nanosheet (≈ 5 nm)	Zn^2+^ ion insertion/extraction	0.2–1.8	1.0/0.8 and 0.6/0.5	489 mAh g^−1^ (100 mA g^−1^)
MnO_2_H_0.16_(H_2_O)_0.27_ (MON)^[^ ^93^ ^]^	Nanosheet (≈2.5 nm)	H^+^/Zn^2+^ ion insertion/extraction	1–1.8	1.55/1.6 and 1.21/1.37	300 mAh g^−1^ (0.1C)
VS_2_@SS^[^ ^99^ ^]^	Hierarchical flower‐like on stainless steel	Zn^2+^ ion insertion/extraction phase transition of VS_2_	0.4–1	0.78/0.69 and 0.69/0.58	187 mAh g^−1^ (100 mA g^−1^)
VS_2_^[^ ^99^ ^]^	Commercial bulk	–	0.4–1	–	117 mAh g^−1^ (100 mA g^−1^)
Na*_x_*V_2_O_5_·*n*H_2_O/RGO/CNT^[^ ^101^ ^]^	Freestanding nanobelt/3D cross‐linked conductive carbonaceous hierarchical structure	Multistep Zn^2+^ ion insertion/extraction	0.2–1.6	1.4/1.1/1/0.7/0.6 and 1.35/0.95/0.8/0.6/0.35	459 mAh g^−1^ (500 mA g^−1^)
Hydrophilic composite cathode (HCC)‐V_3_S_4_^[^ ^102^ ^]^	Nanospherical/composite carbon heterostructure	Zn^2+^ ion insertion/extraction	0.5–1.5	1.15 and 0.9	174 mAh g^−1^ (100 mA g^−1^)
MnO_2_/RGO^[^ ^88^ ^]^	Nanosheet/RGO heterostructure	H^+^ and Zn^2+^ insertion/extraction	1.0–1.9	1.55/1.6 and 1.26/1.39	332.2 mAh g^−1^ (300 mA g^−1^)
MnO_2_^[^ ^88^ ^]^	Nanosheet	H^+^ and Zn^2+^ insertion/extraction	1–1.9	1.55/1.6 and 1.26/1.39	259.1 mAh g^−1^ (mA g^−1^)
MoS_2_@CC^[^ ^22^ ^]^	Nanosheet/carbon cloth	Zn^2+^ insertion/extraction	0.3–1.5	1.28 and 0.64	202.6 mAh g^−1^ (mA g^−1^)
MnO_2_/CC^[^ ^26^ ^]^	Nanosheet‐assembled hollow polyhedron/carbon cloth	H^+^ and Zn^2+^ insertion/extraction	0.8–1.8	1.61/1.38 and 1.38/1.16	350 mAh g^−1^ (100 mA g^−1^)
MnO_2_/CC^[^ ^26^ ^]^	Commercial bulk	–	0.8–1.8	1.61/1.38 and 1.38/1.16	212.8 mAh g^−1^ (100 mA g^−1^)
V_2_O_5_^[^ ^25^ ^]^	Hollow spheres	Multistep Zn^2+^ ion insertion/extraction	0.2–1.5	1.08/0.81 and 0.85/0.56	314 mAh g^−1^ (100 mA g^−1^)
V_2_O_5_^[^ ^25^ ^]^	Commercial bulk	–	0.2–1.5	–	225 mAh g^−1^ (100 mA g^−1^)
V_2_O_5_^[^ ^112^ ^]^	Yolk–shell	H^+^ and Zn^2+^ insertion/extraction	0.4–1.4	0.97/0.62 and 0.98/0.97	410 mAh g^−1^ (100 mA g^−1^)
V_2_O_5_^[^ ^113^ ^]^	Porous microsphere	Zn^2+^ insertion/extraction	0.3–1.5	0.99/0.71 and 0.89/0.55	219 mAh g^−1^ (100 mA g^−1^)

## 4 Electrolytes for Aqueous ZIBs

It is worth mentioning that Zn^2+^ is usually present in hydrated Zn^2+^ form with water molecules surrounded in the water medium. The strong interaction between the Zn^2+^ and water molecules induces a high desolvation and deposition energy barrier for a solvated Zn^2+^.^[^ ^125^ ^]^ The side product of a hydroxyl ion by water decomposition usually leads to the formation of Zn(OH)_2_, which further transforms into insoluble and electrochemically inactive ZnO. The nature of the zinc salt electrolyte has been reported to exhibit significant effects on the stability and electrochemical properties of ZIBs. Therefore, the exploration of suitable electrolytes is critical to control the Zn^2+^ reaction kinetics at the electrode/electrolyte interface.

Since the first use of a mild acidic or neutral electrolyte containing Zn^2+^ for ZIB application, such as ZnSO_4_ or Zn(NO_3_)_2_, a series of other zinc salts including Zn(CH_3_COO)_2_, ZnF_2_, Zn(ClO_4_)_2_, ZnCl_2_, Zn(TFSI)_2_, and Zn(CF_3_SO_3_)_2_ have been then explored for achieving high‐performance aqueous ZIBs.^[^ ^14^, ^15^, ^126^, ^127^, ^128^ ^]^ Although a mild acidic or neutral liquid electrolytes exhibit several advantages, some critical issues such as water‐induced side reactions, uncontrollable solid–liquid interface reactions, cost, and corrosion‐induced instability effects have greatly limited their practical applications for ZIBs. **Table** **3** summarizes the typical aqueous electrolytes for ZIBs in terms of their advantages and disadvantages based on previously reported works.^[^ ^14^, ^15^, ^126^, ^127^, ^128^ ^]^ Overall, the types of zinc salt in the neutral electrolyte are likely to affect the overall zinc storage properties for ZIBs such as operating electrochemical potential window, reversible electrochemical reaction, and the reaction kinetics for Zn deposition and dissolution.

**Table 3:** Comparison of the widely investigated aqueous electrolytes for ZIBs.^[^ ^14^, ^15^, ^126^, ^127^, ^128^ ^]^

Typical electrolytes	Advantages	Disadvantages
ZnSO_4_	Low cost, high solubility in water, high stability, remarkable Zn storage performance, and O_2_ evolution are significantly suppressed up to 1 m ZnSO_4_	Formation of basic zinc sulfates, Zn_4_(OH)_6_SO_4_·*n*H_2_O
Zn(NO_3_)_2_	Rapid and reversible electrochemical dissolution/deposition process, low cost	A narrow electrochemical window, irreversible Zn dissolution/precipitation, strong oxidant effect of nitrate ions will lead to the degradations of both Zn anode and Cu hexacyanoferrate (HCF) cathode
ZnCl_2_	Highly soluble in water, cheaper, high Coulombic efficiency can be achieved with high concentration	A narrow electrochemical window at low concentration, irreversible Zn dissolution/precipitation, instability of halogen ions
Zn(ClO_4_)_2_	Cl^−^ containing layer inhibits the side reaction, enables a stable and rapid Zn deposition and dissolution	A higher overpotential
Zn(CF_3_SO_3_)_2_	High reversibility, fast kinetics for Zn deposition and dissolution, alleviate the cathode dissolution effect, enhance the stability of Zn anode, and reduce solvation effect of Zn ions, wide electrochemical window, parasitical reactions of O_2_ evolution are significantly suppressed up to 2.4 and 2.3 V for 1 m Zn(CF_3_SO_3_)_2_	High cost

Besides the types of zinc salt, salt concentration and the choice of solvent are also revealed to have significant effects on the interfacial (de)solvation kinetics at the electrode–electrolyte interface.^[^ ^125^, ^129^, ^130^ ^]^ Zhang et al. revealed that a better electrochemical performance is observed for the same electrolyte with a higher concentration, including enhanced cycling stability, widen operating potential window, improved specific capacity, and higher Coulombic efficiency of active ions.^[^ ^129^ ^]^ The obtained results can be attributed to the formation of an effective electrode–electrolyte interface with suppressed dendrite growth and negligible water‐induced activity by an ultrahigh concentrated electrolyte. Recently, the “water in salt” concept that employs an ultrahigh concentration zinc salt solution has been introduced into ZIBs by Wang et al.^[^ ^125^ ^]^ By achieving a pH neutral value via manipulating the electrolyte concentration, dendrite‐free cycling of a Zn anode with a 20 m LiTFSI + 1 m Zn(TFSI)_2_ mixture in water–electrolyte was demonstrated. They further revealed that at LiTFSI concentration of more than 20 m, the TFSI^−^ can alter the Zn solvation behavior by eliminating the water molecules around Zn^2+^, which not only promote the electrochemical stability and kinetics but also inhibit the water‐induced side reactions. However, the large voltage polarization induced by the concentrated electrolyte and the expensive Li/Zn salts with fluorinated anions has limited their practical applications. In addition, Han et al. further revealed that zinc electrolyte with organic solvents such as acetonitrile–Zn(TFSI)_2_, acetonitrile–Zn(CF_3_SO_3_)_2_, and propylene carbonate–Zn(TFSI)_2_ electrolytes can provide better anodic stability (up to ≈3.8 V vs Zn/Zn^2+^) and enable highly reversible Zn deposition behavior on a Zn metal anode.^[^ ^130^ ^]^ They demonstrated that types of solvent exhibited significant effect in determining the anodic stability of most zinc salt electrolytes except for Zn(TFSI)_2_.

Other approach such as the introduction of additives has been widely employed to inhibit the Zn dendrite formation and the cathode dissolution effect.^[^ ^50^, ^131^ ^]^ It is widely reported that preaddition of metal ion salts such as Na_2_SO_4_, MnSO_4_, etc., in aqueous ZnSO_4_ electrolyte can promote the reversibility of the cathodic reaction and reduce the cathode dissolution effect.^[^ ^50^, ^131^ ^]^ Recently, Song et al. have further employed various imidazolium ionic liquids as the additives for the ammoniacal electrolytes.^[^ ^131^ ^]^ They revealed that imidazolium ionic liquid additives with different anions can coordinate with metal ions to form the new reduced species to alter the electrode/electrolyte interfacial properties, thereby directly regulate the growth of dendritic zinc, and improve the formation of compact deposits. Nonetheless, the addition of these additives will greatly alter the cathode–electrolyte interfacial reaction and the electrochemical reactions, leading to a series of complex electrochemical reactions and may induce unwanted side reactions that worsen their performance.

## 5 Summary

In summary, aqueous ZIBs have been considered as promising alternatives for large‐scale energy storage systems. Despite the aforementioned achievements, many challenges still exist in aqueous ZIB applications. This review presents the issues in searching appropriate cathode materials for ZIBs, including the lack of suitable Zn^2+^ insertion/extraction cathode materials and controversial reaction mechanisms. Also, we summarize the recent progress in the microstructure engineering of cathode materials for ZIBs and classify them into crystalline structure optimization, crystal defect engineering, interlayer engineering, and morphology engineering. The recent exciting research advances on the investigation of various cathode materials for aqueous ZIBs have demonstrated their great potential in developing large‐scale energy storage systems. Nonetheless, the research field is still in its infancy, and there are still many fundamental issues that need to be tackled toward advanced cathode materials for ZIBs. Therefore, future efforts should be employed for the following aspects to pursuing advanced cathode materials for ZIBs, as discussed below.

First, the cathode electrolyte interface remains a key factor that limited the practical development of ZIBs. In ZIBs, the interface between the electrode and electrolyte is important in determining the electrochemical stability window and governing the overall reaction mechanisms. Most of the current research has been focusing on the exploration of suitable electrolyte with wide electrochemical stability window and little water‐induced side reactions to mitigate interface reaction. Owing to the complexities of cathode electrolyte interface composition and structure that are readily influenced by the type of electrolyte, concentration of electrolyte, additives, and electrochemical measurement condition, the interface issues such as interfacial contact and chemical compatibility of the electrolyte with both electrodes remains a great obstacle for the development of ZIBs. A deep understanding of cathode electrolyte interface formation and evolution based on the cathode properties is critical for the interface design of high voltage ZIBs.

Second, as defect engineering has been demonstrated as a promising approach for modulating the electrochemical activity of cathode materials for ZIBs, more in‐depth characterization techniques such as in situ microscopic techniques (e.g., in situ transimission electron microscopy (TEM) and scanning tunneling microscope (STM) are required for understanding the reaction pathway and the interactions between defects and Zn^2+^ during cycling. Nonetheless, existing research works usually simply characterize the existence of defects and then propose an outstanding effect on electrochemical performance. It is well‐known that some defects are not always stable under practical conditions, resulting in unsatisfactory durability. The development of advanced in situ characterization enables real‐time monitoring of the defect condition during the charge/discharge process, which helps researchers to establish the fundamental relationship of defect performance. Moreover, synthesis methodology with controllable defect species and concentration is essential to realize defect functionalization and obtain a fundamental understanding of the defect‐driven mechanism.

Third, considering the promising potential of layered materials as cathode materials for ZIBs, guidelines on the selection of suitable intercalated foreign species for obtaining appropriate interlayer expansion and promoting device performance are yet to be established. Previous studies have demonstrated that metal‐ion intercalation, such as Li^+^, Na^+^, K^+^, Mg^2+^, and Zn^2+^, can act as pillars to improve the electrochemical performance of layered vanadium oxide cathode materials.^[^ ^77^, ^78^, ^79^, ^80^, ^81^ ^]^ However, the layered vanadium oxide cathode materials still suffer from structural degradation due to the large molecular weight and the volume of metal ions upon repeated insertion and extraction of zinc ions. Therefore, more efforts shall focus on overcoming the induced‐structural degradation effects of metal‐ion intercalation effect by introducing the cointercalation of alkaline ions together with inactive small molecules and optimizing the electrolyte composition. Also, the intercalation and degradation mechanism of metal‐ion intercalation in the host deserves much attention. Recently, the insertion of other neutral molecules or clusters such as polymeric materials has been investigated to modulate the interlayer spacing, which can avoid phase transition and provide stability to the extended interlayer structure for ZIBs. Nonetheless, the insertion of neutral molecules is still relatively less reported and should be further explored.

Fourth, rational microstructure engineering has provided structural benefits such as high surface to mass/volume ratio, improved conductivity, intensive chemical active sites, and stable framework and overcomes the intrinsic issues of bulk materials, such as electronic conductivity and structural stability, rendering ZIBs with high capacity, and stable cycling.  However, most of these achievements were obtained with relatively low areal loading of cathode material (<1 mg cm^−2^). To pave the way to practically viable ZIBs, the development of advanced self‐standing cathodes with an industrial‐level areal capacity (≈35 mAh cm^−2^), superior gravimetric capacity, excellent rate, and cycling stability simultaneously is essential.^[^ ^124^ ^]^ With the increase of cathode loading, electrode–electrolyte interfacial issues that are possibly incubative under low loading may rise. More comprehensive works shall be employed to explore strategies toward advanced high‐loading ZIBs.

Finally, the existence of zinc dendrite in the neutral electrolyte is remaining a great challenge to the practical application of aqueous ZIBs. Many approaches have been devoted to eliminating dendrites such as liquid electrolyte composition optimization, solid‐state electrolyte, and electrode design.^[^ ^132^, ^133^ ^]^ Specifically, the use of ultrahigh concentrated electrolyte with organic anions and organic cosolvent as additives has been reported to effectively suppress the dendrite deposition of zinc metal.^[^ ^125^ ^]^ However, the side effects of decayed H^+^ storage capacity still need more enhancement. Solid‐state electrolytes such as poly(ethylene oxide) (PEO)–ZnX_2_ complexes (X = Cl, Br, I, ClO_4_, and CF_3_SO_3_),^[^ ^134^ ^]^ poly(vinylidene fluoride)/PEO–Zn(CF_3_SO_3_)_2_,^[^ ^135^ ^]^ etc., have been introduced to physically shield dendrite growth but the low electronic conductivity in solid‐state electrolyte induced huge solid–solid interface impedance, which reduces the rate capability of ZIBs. Electrode interface designs such as the electrode surface coating^[^ ^136^, ^137^ ^]^ and application of 3D Zn hosts with conducting graphitic network,^[^ ^138^, ^139^ ^]^ as well as low‐current‐densities‐controlled electrohealing methodology^[^ ^132^ ^]^ with slow galvanostatic discharge/charge appear to be economical approaches to eliminate the formation of zinc dendrite without degrading battery performance.^[^ ^132^ ^]^ Nonetheless, the practical parameters such as current density, stripped and/or deposited capacity, and the interelectrode distance, which can significantly manipulate the Zn cycling performance, were usually not taken into consideration. Therefore, much attention shall be focused to consider and evaluate the experimental conditions based on the practical conditions for achieving commercial large‐scale storage applications.

## Conflict of Interest

The authors declare no conflict of interest.

## References

[1] J. M. Tarascon , M. Armand , Nature 2001, 414, 359. 11713543 10.1038/35104644

[2] N. Nitta , F. Wu , J. T. Lee , G. Yushin , Mater. Today 2015, 18, 252.

[3] M. A. Hannan , M. S. H. Lipu , A. Hussain , A. Mohamed , Renewable Sustainable Energy Rev. 2017, 78, 834.

[4] Y. Fang , L. Xiao , Z. Chen , X. Ai , Y. Cao , H. Yang , Electrochem. Energy Rev. 2018, 1, 294.

[5] K. Liu , Y. Liu , D. Lin , A. Pei , Y. Cui , Sci. Adv. 2018, 4, eaas9820. 29942858 10.1126/sciadv.aas9820 PMC6014713

[6] Z. P. Cano , D. Banham , S. Ye , A. Hintennach , J. Lu , M. Fowler , Z. Chen , Nat. Energy 2018, 3, 279.

[7] C. Xu , B. Li , H. Du , F. Kang , Angew. Chem., Int. Ed. 2012, 51, 933. 10.1002/anie.201106307 22170816

[8] M.‐C. Lin , M. Gong , B. Lu , Y. Wu , D.‐Y. Wang , M. Guan , M. Angell , C. Chen , J. Yang , B.‐J. Hwang , H. Dai , Nature 2015, 520, 324. 10.1038/nature14340 25849777

[9] D. Selvakumaran , A. Pan , S. Liang , G. Cao , J. Mater. Chem. A 2019, 7, 18209.

[10] G. Fang , J. Zhou , A. Pan , S. Liang , ACS Energy Lett. 2018, 3, 2480.

[11] F. Wan , Z. Niu , Angew. Chem., Int. Ed. 2019, 58, 16358. 10.1002/anie.201903941 31050086

[12] C. Li , X. Xie , S. Liang , J. Zhou , Energy Environ. Mater. 2020, 3, 146

[13] J. Ming , J. Guo , C. Xia , W. Wang , H. N. Alshareef , Mater. Sci. Eng., R 2019, 135, 58.

[14] M. Song , H. Tan , D. Chao , H. J. Fan , Adv. Funct. Mater. 2018, 28, 1802564.

[15] Y. Zhao , Y. Zhu , X. Zhang , InfoMat 2020, 2, 237.

[16] B. Tang , L. Shan , S. Liang , J. Zhou , Energy Environ. Sci. 2019, 12, 3288.

[17] W. Xu , C. Sun , K. Zhao , X. Cheng , S. Rawal , Y. Xu , Y. Wang , Energy Storage Mater. 2019, 16, 527.

[18] T. Xiong , Z. G. Yu , H. Wu , Y. Du , Q. Xie , J. Chen , Y.‐W. Zhang , S. J. Pennycook , W. S. V. Lee , J. Xue , Adv. Energy Mater. 2019, 9, 1803815.

[19] C. Zhu , G. Fang , S. Liang , Z. Chen , Z. Wang , J. Ma , H. Wang , B. Tang , X. Zheng , J. Zhou , Energy Storage Mater. 2020, 24, 394.

[20] Y. Lu , J. Wang , S. Zeng , L. Zhou , W. Xu , D. Zheng , J. Liu , Y. Zeng , X. Lu , J. Mater. Chem. A 2019, 7, 21678.

[21] J. Lai , H. Zhu , X. Zhu , H. Koritala , Y. Wang , ACS Appl. Energy Mater. 2019, 2, 1988.

[22] H. Li , Q. Yang , F. Mo , G. Liang , Z. Liu , Z. Tang , L. Ma , J. Liu , Z. Shi , C. Zhi , Energy Storage Mater. 2019, 19, 94.

[23] X. Li , M. Li , Q. Yang , H. Li , H. Xu , Z. Chai , K. Chen , Z. Liu , Z. Tang , L. Ma , Z. Huang , B. Dong , X. Yin , Q. Huang , C. Zhi , ACS Nano 2020, 14, 541. 31917537 10.1021/acsnano.9b06866

[24] Q. Yang , Z. Huang , X. Li , Z. Liu , H. Li , G. Liang , D. Wang , Q. Huang , S. Zhang , S. Chen , C. Zhi , ACS Nano 2019, 13, 8275. 31244041 10.1021/acsnano.9b03650

[25] L. Chen , Z. Yang , F. Cui , J. Meng , H. Chen , X. Zeng , Appl. Surf. Sci. 2020, 507, 145137.

[26] F. Wu , X. Gao , X. Xu , Y. Jiang , X. Gao , R. Yin , W. Shi , W. Liu , G. Lu , X. Cao , ChemSusChem 2020, 13, 1537. 31797574 10.1002/cssc.201903006

[27] H. Liang , Z. Cao , F. Ming , W. Zhang , D. H. Anjum , Y. Cui , L. Cavallo , H. N. Alshareef , Nano Lett. 2019, 19, 3199. 30986352 10.1021/acs.nanolett.9b00697

[28] W. Liu , J. Hao , C. Xu , J. Mou , L. Dong , F. Jiang , Z. Kang , J. Wu , B. Jiang , F. Kang , Chem. Commun. 2017, 53, 6872. 10.1039/c7cc01064h 28604865

[29] G. Fang , C. Zhu , M. Chen , J. Zhou , B. Tang , X. Cao , X. Zheng , A. Pan , S. Liang , Adv. Funct. Mater. 2019, 29, 1808375.

[30] H. Pan , Y. Shao , P. Yan , Y. Cheng , K. S. Han , Z. Nie , C. Wang , J. Yang , X. Li , P. Bhattacharya , K. T. Mueller , J. Liu , Nat. Energy 2016, 1, 16039.

[31] N. Zhang , F. Cheng , J. Liu , L. Wang , X. Long , X. Liu , F. Li , J. Chen , Nat. Commun. 2017, 8, 405. 28864823 10.1038/s41467-017-00467-x PMC5581336

[32] P. Oberholzer , E. Tervoort , A. Bouzid , A. Pasquarello , D. Kundu , ACS Appl. Mater. Interfaces 2019, 11, 674. 30521309 10.1021/acsami.8b16284

[33] J. H. Jo , Y.‐K. Sun , S.‐T. Myung , J. Mater. Chem. A 2017, 5, 8367.

[34] G. Fang , S. Liang , Z. Chen , P. Cui , X. Zheng , A. Pan , B. Lu , X. Lu , J. Zhou , Adv. Funct. Mater. 2019, 29, 1905267.

[35] F. Wan , Y. Zhang , L. Zhang , D. Liu , C. Wang , L. Song , Z. Niu , J. Chen , Angew. Chem., Int. Ed. 2019, 58, 7062. 10.1002/anie.201902679 30893503

[36] C. Wei , C. Xu , B. Li , H. Du , F. Kang , J. Phys. Chem. Solids 2012, 73, 1487.

[37] B. Lee , C. S. Yoon , H. R. Lee , K. Y. Chung , B. W. Cho , S. H. Oh , Sci. Rep. 2015, 4, 6066. 10.1038/srep06066 PMC5377529 25317571

[38] B. Lee , H. R. Lee , H. Kim , K. Y. Chung , B. W. Cho , S. H. Oh , Chem. Commun. 2015, 51, 9265. 10.1039/c5cc02585k 25920416

[39] M. H. Alfaruqi , V. Mathew , J. Gim , S. Kim , J. Song , J. P. Baboo , S. H. Choi , J. Kim , Chem. Mater. 2015, 27, 3609.

[40] Y. Yuan , C. Zhan , K. He , H. Chen , W. Yao , S. Sharifi‐Asl , B. Song , Z. Yang , A. Nie , X. Luo , H. Wang , S. M. Wood , K. Amine , M. S. Islam , J. Lu , R. Shahbazian‐Yassar , Nat. Commun. 2016, 7, 13374. 27869120 10.1038/ncomms13374 PMC5473628

[41] D. A. Tompsett , S. C. Parker , M. S. Islam , J. Am. Chem. Soc. 2014, 136, 1418. 24446882 10.1021/ja4092962

[42] K. Zhang , X. Han , Z. Hu , X. Zhang , Z. Tao , J. Chen , Chem. Soc. Rev. 2015, 44, 699. 25200459 10.1039/c4cs00218k

[43] N. Zhang , Y. Dong , M. Jia , X. Bian , Y. Wang , M. Qiu , J. Xu , Y. Liu , L. Jiao , F. Cheng , ACS Energy Lett. 2018, 3, 1366.

[44] L. Zhang , L. Chen , X. Zhou , Z. Liu , Adv. Energy Mater. 2015, 5, 1400930.

[45] M. S. Chae , J. W. Heo , H. H. Kwak , H. Lee , S.‐T. Hong , J. Power Sources 2017, 337, 204.

[46] G. Li , Z. Yang , Y. Jiang , C. Jin , W. Huang , X. Ding , Y. Huang , Nano Energy 2016, 25, 211.

[47] H. B. Zhao , C. J. Hu , H. W. Cheng , J. H. Fang , Y. P. Xie , W. Y. Fang , T. N. L. Doan , T. K. A. Hoang , J. Q. Xu , P. Chen , Sci. Rep. 2016, 6, 25809. 27174224 10.1038/srep25809 PMC4865945

[48] Y. Huang , J. Mou , W. Liu , X. Wang , L. Dong , F. Kang , C. Xu , Nano‐Micro Lett. 2019, 11, 49. 10.1007/s40820-019-0278-9 PMC7770901 34138004

[49] W. Sun , F. Wang , S. Hou , C. Yang , X. Fan , Z. Ma , T. Gao , F. Han , R. Hu , M. Zhu , C. Wang , J. Am. Chem. Soc. 2017, 139, 9775. 28704997 10.1021/jacs.7b04471

[50] F. Wan , L. Zhang , X. Dai , X. Wang , Z. Niu , J. Chen , Nat. Commun. 2018, 9, 1656. 29695711 10.1038/s41467-018-04060-8 PMC5916908

[51] T. R. Juran , J. Young , M. Smeu , J. Phys. Chem. C 2018, 122, 8788.

[52] S. H. Kim , S. M. Oh , J. Power Sources 1998, 72, 150.

[53] S. Islam , M. H. Alfaruqi , V. Mathew , J. Song , S. Kim , S. Kim , J. Jo , J. P. Baboo , D. T. Pham , D. Y. Putro , Y.‐K. Sun , J. Kim , J. Mater. Chem. A 2017, 5, 23299.

[54] J. Lee , J. B. Ju , W. I. Cho , B. W. Cho , S. H. Oh , Electrochim. Acta 2013, 112, 138.

[55] C. Yuan , Y. Zhang , Y. Pan , X. Liu , G. Wang , D. Cao , Electrochim. Acta 2014, 116, 404.

[56] D. Kundu , P. Oberholzer , C. Glaros , A. Bouzid , E. Tervoort , A. Pasquarello , M. Niederberger , Chem. Mater. 2018, 30, 3874.

[57] Y. Cheng , L. Luo , L. Zhong , J. Chen , B. Li , W. Wang , S. X. Mao , C. Wang , V. L. Sprenkle , G. Li , J. Liu , ACS Appl. Mater. Interfaces 2016, 8, 13673. 27182714 10.1021/acsami.6b03197

[58] T. Wei , Q. Li , G. Yang , C. Wang , J. Mater. Chem. A 2018, 6, 8006.

[59] B. Tang , J. Zhou , G. Fang , S. Guo , X. Guo , L. Shan , Y. Tang , S. Liang , J. Electrochem. Soc. 2019, 166, A480.

[60] J. Ding , Z. Du , L. Gu , B. Li , L. Wang , S. Wang , Y. Gong , S. Yang , Adv. Mater. 2018, 30, 1800762. 10.1002/adma.201800762 29761561

[61] L. Shan , J. Zhou , W. Zhang , C. Xia , S. Guo , X. Ma , G. Fang , X. Wu , S. Liang , Energy Technol. 2019, 7, 1900022.

[62] J. Ding , Z. Du , B. Li , L. Wang , Y. Gong , S. Yang , Adv. Mater. 2019, 31, 1904369. 10.1002/adma.201904369 31538380

[63] Q. Zhao , W. Huang , Z. Luo , L. Liu , Y. Lu , Y. Li , L. Li , J. Hu , H. Ma , J. Chen , Sci. Adv. 2018, 4, eaao1761. 29511734 10.1126/sciadv.aao1761 PMC5837429

[64] B. Haupler , C. Rossel , A. M. Schwenke , J. Winsberg , D. Schmidt , A. Wild , U. S. Schubert , NPG Asia Mater. 2016, 8, e283.

[65] R. Y. Wang , C. D. Wessells , R. A. Huggins , Y. Cui , Nano Lett. 2013, 13, 5748. 24147617 10.1021/nl403669a

[66] Q. Yang , F. Mo , Z. Liu , L. Ma , X. Li , D. Fang , S. Chen , S. Zhang , C. Zhi , Adv. Mater. 2019, 31, 1901521. 10.1002/adma.201901521 31192499

[67] J. S. Ko , P. P. Paul , G. Wan , N. Seitzman , R. H. DeBlock , B. S. Dunn , M. F. Toney , J. Nelson Weker , Chem. Mater. 2020, 32, 3028.

[68] M. S. Chae , J. W. Heo , S.‐C. Lim , S.‐T. Hong , Inorg. Chem. 2016, 55, 3294. 26967205 10.1021/acs.inorgchem.5b02362

[69] M. Han , J. Huang , S. Liang , L. Shan , X. Xie , Z. Yi , Y. Wang , S. Guo , J. Zhou , iScience 2020, 23, 100797. 31927485 10.1016/j.isci.2019.100797 PMC6957857

[70] W. Yang , L. Dong , W. Yang , C. Xu , G. Shao , G. Wang , Small Methods 2020, 4, 1900670.

[71] M. Liao , J. Wang , L. Ye , H. Sun , Y. Wen , C. Wang , X. Sun , B. Wang , H. Peng , Angew. Chem., Int. Ed. 2020, 59, 2273. 10.1002/anie.201912203 31743581

[72] Z. Li , Y. Ren , L. Mo , C. Liu , K. Hsu , Y. Ding , X. Zhang , X. Li , L. Hu , D. Ji , G. Cao , ACS Nano 2020, 14, 5581. 32392033 10.1021/acsnano.9b09963

[73] J. C. Knight , S. Therese , A. Manthiram , J. Mater. Chem. A 2015, 3, 21077.

[74] N. Zhang , F. Cheng , Y. Liu , Q. Zhao , K. Lei , C. Chen , X. Liu , J. Chen , J. Am. Chem. Soc. 2016, 138, 12894. 27627103 10.1021/jacs.6b05958

[75] J. Xu , J. Zhang , W. Zhang , C.‐S. Lee , Adv. Energy Mater. 2017, 7, 1700571.

[76] C. Liu , Z. Neale , J. Zheng , X. Jia , J. Huang , M. Yan , M. Tian , M. Wang , J. Yang , G. Cao , Energy Environ. Sci. 2019, 12, 2273.

[77] M. Yan , P. He , Y. Chen , S. Wang , Q. Wei , K. Zhao , X. Xu , Q. An , Y. Shuang , Y. Shao , K. T. Mueller , L. Mai , J. Liu , J. Yang , Adv. Mater. 2018, 30, 1703725. 10.1002/adma.201703725 29131432

[78] Y. Yang , Y. Tang , G. Fang , L. Shan , J. Guo , W. Zhang , C. Wang , L. Wang , J. Zhou , S. Liang , Energy Environ. Sci. 2018, 11, 3157.

[79] P. He , G. Zhang , X. Liao , M. Yan , X. Xu , Q. An , J. Liu , L. Mai , Adv. Energy Mater. 2018, 8, 1702463.

[80] S. Li , M. Chen , G. Fang , L. Shan , X. Cao , J. Huang , S. Liang , J. Zhou , J. Alloys Compd. 2019, 801, 82.

[81] C. Xia , J. Guo , P. Li , X. Zhang , H. N. Alshareef , Angew. Chem., Int. Ed. 2018, 57, 3943. 10.1002/anie.201713291 29432667

[82] J. Huang , Z. Wang , M. Hou , X. Dong , Y. Liu , Y. Wang , Y. Xia , Nat. Commun. 2018, 9, 2906. 30046036 10.1038/s41467-018-04949-4 PMC6060179

[83] D. Bin , W. Huo , Y. Yuan , J. Huang , Y. Liu , Y. Zhang , F. Dong , Y. Wang , Y. Xia , Chem 2020, 6, 968.

[84] Y. Liu , Q. Li , K. Ma , G. Yang , C. Wang , ACS Nano 2019, 13, 12081. 31553172 10.1021/acsnano.9b06484

[85] B. Sambandam , V. Soundharrajan , S. Kim , M. H. Alfaruqi , J. Jo , S. Kim , V. Mathew , Y.‐k. Sun , J. Kim , J. Mater. Chem. A 2018, 6, 3850.

[86] P. He , Y. Quan , X. Xu , M. Yan , W. Yang , Q. An , L. He , L. Mai , Small 2017, 13, 1702551. 10.1002/smll.201702551 29152849

[87] X. He , H. Zhang , X. Zhao , P. Zhang , M. Chen , Z. Zheng , Z. Han , T. Zhu , Y. Tong , X. Lu , Adv. Sci. 2019, 6, 1900151. 10.1002/advs.201900151 PMC6662057 31380205

[88] S. Chen , Y. Zhang , H. Geng , Y. Yang , X. Rui , C. C. Li , J. Power Sources 2019, 441, 227192.

[89] X. Li , L. Ma , Y. Zhao , Q. Yang , D. Wang , Z. Huang , G. Liang , F. Mo , Z. Liu , C. Zhi , Mater. Today Energy 2019, 14, 100361.

[90] J. Lai , H. Tang , X. Zhu , Y. Wang , J. Mater. Chem. A 2019, 7, 23140.

[91] K. Zhu , T. Wu , K. Huang , ACS Nano 2019, 13, 14447. 31765124 10.1021/acsnano.9b08039

[92] Y. Huang , J. Liu , Q. Huang , Z. Zheng , P. Hiralal , F. Zheng , D. Ozgit , S. Su , S. Chen , P.‐H. Tan , S. Zhang , H. Zhou , npj Flexible Electron. 2018, 2, 21.

[93] F. Ming , H. Liang , Y. Lei , S. Kandambeth , M. Eddaoudi , H. N. Alshareef , ACS Energy Lett. 2018, 3, 2602.

[94] B. He , Z. Zhou , P. Man , Q. Zhang , C. Li , L. Xie , X. Wang , Q. Li , Y. Yao , J. Mater. Chem. A 2019, 7, 12979.

[95] H. Ren , J. Zhao , L. Yang , Q. Liang , S. Madhavi , Q. Yan , Nano Res. 2019, 12, 1347.

[96] C. Guo , H. Liu , J. Li , Z. Hou , J. Liang , J. Zhou , Y. Zhu , Y. Qian , Electrochim. Acta 2019, 304, 370.

[97] Q. Zhao , X. Chen , Z. Wang , L. Yang , R. Qin , J. Yang , Y. Song , S. Ding , M. Weng , W. Huang , J. Liu , W. Zhao , G. Qian , K. Yang , Y. Cui , H. Chen , F. Pan , Small 2019, 15, 1904545. 10.1002/smll.201904545 31588653

[98] K. Raju , H. Han , D. B. Velusamy , Q. Jiang , H. Yang , F. P. Nkosi , N. Palaniyandy , K. Makgopa , Z. Bo , K. I. Ozoemena , ACS Energy Lett. 2020, 5, 23.

[99] X. Wang , L. Ma , J. Sun , ACS Appl. Mater. Interfaces 2019, 11, 41297. 31613584 10.1021/acsami.9b13103

[100] Y. Luo , L. Wei , H. Geng , Y. Zhang , Y. Yang , C. C. Li , ACS Appl. Mater. Interfaces 2020, 12, 11753. 32068382 10.1021/acsami.0c00057

[101] M. S. Javed , H. Lei , Z. Wang , B.‐t. Liu , X. Cai , W. Mai , Nano Energy 2020, 70, 104573.

[102] P. He , M. Yan , G. Zhang , R. Sun , L. Chen , Q. An , L. Mai , Adv. Energy Mater. 2017, 7, 1601920.

[103] X. Wang , Y. Li , S. Wang , F. Zhou , P. Das , C. Sun , S. Zheng , Z. S. Wu , Adv. Energy Mater. 2020, 10, 2000081.

[104] T. Jiao , Q. Yang , S. Wu , Z. Wang , D. Chen , D. Shen , B. Liu , J. Cheng , H. Li , L. Ma , C. Zhi , W. Zhang , J. Mater. Chem. A 2019, 7, 16330.

[105] Y. Ding , Y. Peng , S. Chen , X. Zhang , Z. Li , L. Zhu , L.‐E. Mo , L. Hu , ACS Appl. Mater. Interfaces 2019, 11, 44109. 31687795 10.1021/acsami.9b13729

[106] G. Xu , X. Liu , S. Huang , L. Li , X. Wei , J. Cao , L. Yang , P. K. Chu , ACS Appl. Mater. Interfaces 2020, 12, 706. 31799821 10.1021/acsami.9b17653

[107] S. Liu , X. Chen , Q. Zhang , J. Zhou , Z. Cai , A. Pan , ACS Appl. Mater. Interfaces 2019, 11, 36676. 31538766 10.1021/acsami.9b12128

[108] L. Wang , K.‐W. Huang , J. Chen , J. Zheng , Sci. Adv. 2019, 5, eaax4279. 32047853 10.1126/sciadv.aax4279 PMC6984968

[109] S. Khamsanga , R. Pornprasertsuk , T. Yonezawa , A. A. Mohamad , S. Kheawhom , Sci. Rep. 2019, 9, 8441. 31186468 10.1038/s41598-019-44915-8 PMC6560026

[110] C. Zhu , G. Fang , J. Zhou , J. Guo , Z. Wang , C. Wang , J. Li , Y. Tang , S. Liang , J. Mater. Chem. A 2018, 6, 9677.

[111] S. Fan , S. Huang , Y. Chen , Y. Shang , Y. Wang , D. Kong , M. E. Pam , L. Shi , Y. W. Lim , Y. Shi , H. Y. Yang , Energy Storage Mater. 2019, 23, 17.

[112] Y. Pan , K. Sun , S. Liu , X. Cao , K. Wu , W.‐C. Cheong , Z. Chen , Y. Wang , Y. Li , Y. Liu , D. Wang , Q. Peng , C. Chen , Y. Li , J. Am. Chem. Soc. 2018, 140, 2610. 29341596 10.1021/jacs.7b12420

[113] S. Huang , L. Liu , Y. Wang , Y. Shang , L. Zhang , J. Wang , Y. Zheng , O. G. Schmidt , H. Y. Yang , J. Mater. Chem. A 2019, 7, 6651.

[114] G. Zheng , Y. Yang , J. J. Cha , S. S. Hong , Y. Cui , Nano Lett. 2011, 11, 4462. 21916442 10.1021/nl2027684

[115] L. Chen , Z. Yang , F. Cui , J. Meng , Y. Jiang , J. Long , X. Zeng , Mater. Chem. Front. 2020, 4, 213.

[116] H. Yang , X. Wang , Adv. Mater. 2019, 31, 1800743. 10.1002/adma.201800743 30039881

[117] R. Li , H. Zhang , Q. Zheng , X. Li , J. Mater. Chem. A 2020, 8, 5186.

[118] P. Hu , T. Zhu , J. Ma , C. Cai , G. Hu , X. Wang , Z. Liu , L. Zhou , L. Mai , Chem. Commun. 2019, 55, 8486. 10.1039/c9cc04053f 31268100

[119] F. Xie , L. Zhang , C. Ye , M. Jaroniec , S.‐Z. Qiao , Adv. Mater. 2019, 31, 1800492. 10.1002/adma.201800492 29971832

[120] Z. Li , Y. Fang , J. Zhang , X. W. Lou , Adv. Mater. 2018, 30, 1800525. 10.1002/adma.201800525 29920788

[121] C. Wu , X. Tong , Y. Ai , D.‐S. Liu , P. Yu , J. Wu , Z. M. Wang , Nano‐Micro Lett. 2018, 10, 40. 10.1007/s40820-018-0194-4 PMC6199087 30393689

[122] J. Wang , Y. Cui , D. Wang , Adv. Mater. 2019, 31, 1801993.

[123] F. Liu , Z. Chen , G. Fang , Z. Wang , Y. Cai , B. Tang , J. Zhou , S. Liang , Nano‐Micro Lett. 2019, 11, 25. 10.1007/s40820-019-0256-2 PMC7770672 34137986

[124] D. Chao , W. Zhou , F. Xie , C. Ye , H. Li , M. Jaroniec , S. Z. Qiao , Sci. Adv. 2020, 6, eaba4098. 32494749 10.1126/sciadv.aba4098 PMC7244306

[125] F. Wang , O. Borodin , T. Gao , X. Fan , W. Sun , F. Han , A. Faraone , J. A. Dura , K. Xu , C. Wang , Nat. Mater. 2018, 17, 543. 29662160 10.1038/s41563-018-0063-z

[126] H. Jia , Z. Wang , B. Tawiah , Y. Wang , C. Y. Chan , B. Fei , F. Pan , Nano Energy 2020, 70, 104523.

[127] H. Li , L. Ma , C. Han , Z. Wang , Z. Liu , Z. Tang , C. Zhi , Nano Energy 2019,62, 550.

[128] L. E. Blanc , D. Kundu , L. F. Nazar , Joule 2020, 4, 771.

[129] H. Zhang , F. Cheng , Y. Liu , Q. Zhao , K. Lei , C. Chen , X. Liu , J. Chen , J. Am. Chem. Soc. 2016, 138, 12894. 27627103 10.1021/jacs.6b05958

[130] S. D. Han , N. N. Rajput , X. Qu , B. Pan , M. He , M. S. Ferrandon , C. Liao , K. A. Persson , A. K. Burrell , ACS Appl. Mater. Interfaces 2016, 8, 3021. 26765789 10.1021/acsami.5b10024

[131] Y. Song , J. Hu , J. Tang , W. Gu , L. He , X. Ji , ACS Appl. Mater. Interfaces 2016, 8, 32031. 27933970 10.1021/acsami.6b11098

[132] Q. Yang , G. Liang , Y. Guo , Z. Liu , B. Yan , D. Wang , Z. Huang , X. Li , J. Fan , C. Zhi , Adv. Mater. 2019, 31, 1903778. 10.1002/adma.201903778 31517400

[133] Q. Yang , Y. Guo , B. Yan , C. Wang , Z. Liu , Z. Huang , Y. Wang , Y. Li , H. Li , L. Song , J. Fan , C. Zhi , Adv. Mater. 2020, 32, 2001755. 10.1002/adma.202001755 32406976

[134] A. R. Mainar , E. Iruin , L. C. Colmenares , A. Kvasha , I. de Meatza , M. Bengoechea , O. Leonet , I. Boyano , Z. Zhang , J. A. Blazquez , J. Energy Storage 2018, 15, 304.

[135] R. Rathika , O. Padmaraj , S. A. Suthanthiraraj , Ionics 2018, 24, 243.

[136] H. Li , C. Xu , C. Han , Y. Chen , C. Wei , B. Li , F. Kang , J. Electrochem. Soc. 2015, 162, A1439.

[137] A. Xia , X. Pu , Y. Tao , H. Liu , Y. Wang , Appl. Surf. Sci. 2019, 481, 852.

[138] L. P. Wang , W. N. Li , T. S. Wang , Y. X. Yin , Y. G. Guo , C. R. Wang , Electrochim. Acta 2017, 244, 172.

[139] Y. X. Zeng , X. Y. Zhang , R. F. Qin , X. Q. Liu , P. P. Fang , D. Z. Zheng , Y. X. Tong , X. H. Lu , Adv. Mater. 2019, 31, 1903675. 10.1002/adma.201903675 31342572

